# New Pyrazole-Hydrazone Derivatives: X-ray Analysis, Molecular Structure Investigation via Density Functional Theory (DFT) and Their High In-Situ Catecholase Activity

**DOI:** 10.3390/ijms18112215

**Published:** 2017-10-25

**Authors:** Khalid Karrouchi, El Bekkaye Yousfi, Nada Kheira Sebbar, Youssef Ramli, Jamal Taoufik, Younes Ouzidan, M’hammed Ansar, Yahia N. Mabkhot, Hazem A. Ghabbour, Smaail Radi

**Affiliations:** 1 Laboratoire de Chimie Thérapeutique, Faculté de Médecine et de Pharmacie, Université Mohammed V, P. O. Box 8007, Rabat 10100, Morocco; khalid.karrouchi@um5s.net.ma (K.K.); yramli76@yahoo.fr (Y.R.); jataoufik@hotmail.com (J.T.); ansarmhammed@gmail.com (M.A.); 2 Laboratoire de Chimie Appliquée et Environnement (LCAE), Faculté des Sciences, Université Mohamed I, P. O. Box 524, Oujda 60000, Morocco; 3 Laboratoire National de Contrôle des Médicaments, Direction du Médicament et de la Pharmacie, Ministère de la Santé, P. O. Box 6206, Rabat 10100, Morocco; 4 Institution Supérieure des Professions Infirmières et Techniques de Santé, P. O. Box 4806, Oujda 60000, Morocco; yousfi@netcourrier.com; 5 Laboratoire de Chimie Organique Hétérocyclique, Pharmacochimie, Faculté des sciences, Université Mohammed V, P. O. Box 8007, Rabat 10100, Morocco; snounousebbar@gmail.com; 6 Laboratoire de Chimie Organique Appliquée, Faculté des Sciences et Techniques, Université Sidi Mohamed Ben Abdellah, P. O. Box 2202, Fès 30000, Morocco; younes.ouzidan@usmba.ac.ma; 7 Department of Chemistry, Faculty of Science, King Saud University, P.O. Box 2455, Riyadh 11451, Saudi Arabia; 8 Department of Pharmaceutical Chemistry, College of Pharmacy, King Saud University, P. O. Box 2457, Riyadh 11451, Saudi Arabia; ghabbourh@yahoo.com

**Keywords:** pyrazole, hydrazone, crystal structure, DFT, catecholase activity

## Abstract

The development of low-cost catalytic systems that mimic the activity of tyrosinase enzymes (Catechol oxidase) is of great promise for future biochemistry technologic demands. Herein, we report the synthesis of new biomolecules systems based on hydrazone derivatives containing a pyrazole moiety (**L1**–**L6**) with superior catecholase activity. Crystal structures of **L1** and **L2** biomolecules were determined by X-ray single crystal diffraction (XRD). Optimized geometrical parameters were calculated by density functional theory (DFT) at B3LYP/6–31G (d, p) level and were found to be in good agreement with single crystal XRD data. Copper (II) complexes of the compounds (**L1**–**L6**), generated in-situ, were investigated for their catalytic activities towards the oxidation reaction of catechol to *ortho*-quinone with the atmospheric dioxygen, in an attempt to model the activity of the copper containing enzyme tyrosinase. The studies showed that the activities depend on four parameters: the nature of the ligand, the nature of counter anion, the nature of solvent and the concentration of ligand. The Cu(II)-ligands, given here, present the highest catalytic activity (72.920 μmol·L^−1^·min^−1^) among the catalysts recently reported in the existing literature.

## 1. Introduction

Bioorganometallic compounds are reputed for their remarkable applications in the field of catalysis; however, much less is known about their potential in chemical biology [1,2]. An important goal in organometallic chemistry is the synthesis of biomolecules that exhibit catalytic activity analogous to the activity of enzymes. A number of catalysts having biomimetic activity for different enzymes have been designed by chemists [3,4]. The preparation and etude of efficient template complexes for metalloenzymes with oxidase or oxygenase activity are thus most important for the elaboration of new veritable catalysts for oxidation reactions [5]. Copper ions, as centers of potent site of various metalloproteins, play an essential role in several biological processes: electron transfer, oxidation, transport of dioxygen, etc. [6,7]. Copper complexes of low molecular weight are studied as structural and functional models of active centers of enzymes with copper [8,9,10,11].

Catechol oxidases perform the oxidation of 1,2-diphenols, such as catechol, to *o*-quinones, using dioxygen (O_2_). Hydrogen atoms removed from catechol combine with oxygen to form water. The crystal structures of different forms of catechol oxidase have improved the accordance of the mechanism of catecholase activity of catechol oxidase. Several workers proposed the mechanism of catechol oxidation by natural enzyme, including important proposals by Solomon and kerbs [12,13]. In addition to the high number of Cu(II) complexes reported, their catecholase activity has also been demonstrated, although their mechanistic aspects are not as well understood as those of Cu(II) complexes [14,15,16,17,18].

Hydrazones are members of the Schiff bases family, which are built with aromatic acid hydrazides and carbonyl compounds. They are quite interesting in coordination chemistry as they present a combination of donor sites, such as a protonated/deprotonated amide oxygen atom, imine nitrogen atom of the hydrazone moiety and an additional donor site (usually *N* or *O*) provided from the aldehyde or ketone [19,20]. Hydrazones form wide variety of complexes with chemical, structural, biological and industrial importance [21,22,23]. These proprieties are attributed to the formation of stable chelate complexes with transition metals which catalyze physiological processes [24,25].

**Scheme 1:**
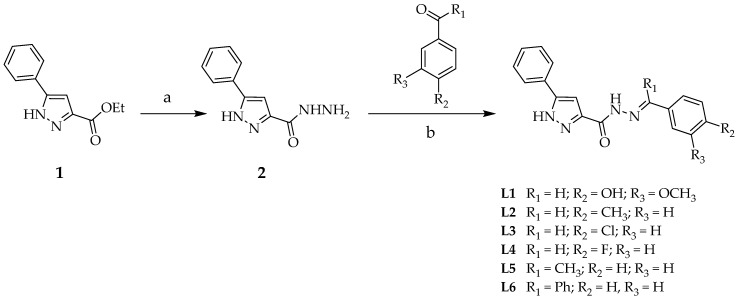
The synthetic routes of compounds **L1**–**L6**: (**a**) hydrazine hydrate (80%), ethanol, reflux 5h; and (**b**) ethanol, acetic acid, reflux, 2–5 h.

In this study, we report the synthesis of six new hydrazone derivatives containing pyrazole moiety with a good yield (Scheme 1). The X-ray crystal structures of compounds **L1** and **L2** were determined, and their geometrical parameters were compared with theoretical DFT calculations at the B3LYP level of theory. The investigation of catalytic activities of copper (II)-ligand complexes towards oxidation of catechol to *o*-quinone was studied. All of the parameters that can affect the catalytic efficiency were studied.

## 2. Results

### 2.1. Synthesis

Synthesis of hydrazones (**L1**–**L6**) is outlined in Scheme 1. Starting compound, ethyl 3-phenyl-*1H*-pyrazole-5-carboxylate (**1**), was readily synthesized by the reaction of ethyl-2,4-dioxo-4-phenyl-butanoate, obtained from acetophenone, and diethyl oxalate, with hydrazine in the presence of sulfuric acid at room temperature. The reaction of ethyl 3-phenyl-*1H*-pyrazole-5-carboxylate (**1**) with hydrazine hydrate in ethanol afforded 3-phenyl-1*H*-pyrazole-5-carbohydrazide (**2**). Finally, the novel desired hydrazones (**L1**–**L6**) were obtained by condensing compound (**2**) with aromatic aldehydes at reflux of ethanol using acetic acid as reported in our previous procedure [26,27,28]. The expected products were isolated as crystalline materials with reliable to excellent yields.

### 2.2. X-Ray Crystal Structures Description

Compounds **L1** and **L2** were analyzed by X-ray diffraction. Refinement parameters and crystal data are listed in Table S1. Supplementary data are deposited with the Cambridge Crystallographic Data Centre (CCDC) under deposition numbers 1522882 and 1523265.

**Figure 1:**
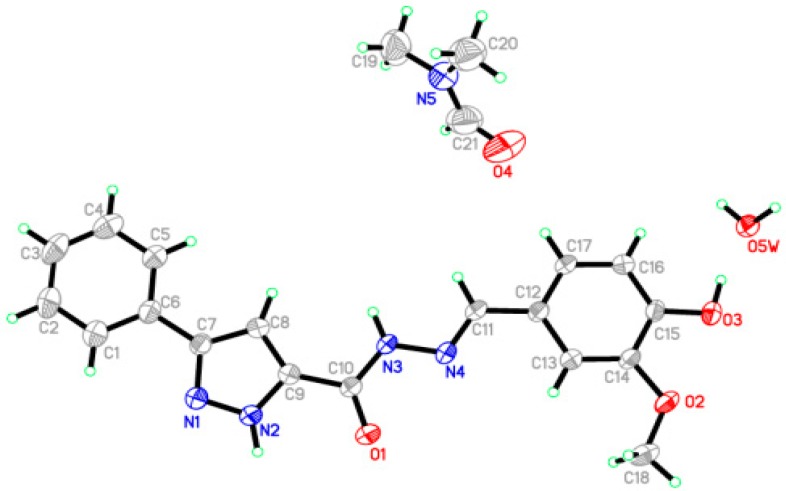
Asymmetric unit of **L1** (CCDC 1522882).

**Figure 2:**
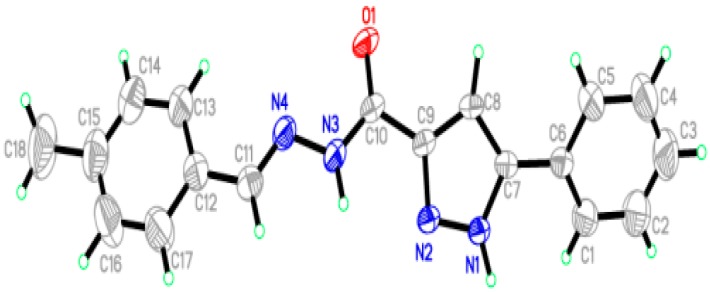
Asymmetric unit of **L2** (CCDC 1523265).

The selected bond lengths and bond angles and hydrogen bonds are listed in Tables S2–S5 in the Supplementary Materials. The asymmetric unit of **L1** contains one independent molecule with DMF and water molecules as mixed crystallizing solvent, as shown in Figure 1. All the bind lengths and angles are in normal ranges. In the crystal wrap, molecules are connected via many classical and non-classical intermolecular hydrogen bonds (Table S3). Figure 2 shows the crystal structure of **L2**. In the crystal structure, the pyrazole ring (N1/N2/C7–C9) makes dihedral angles with the phenyl ring (C1–C6) and tolyl ring (C12–C17), at 23.39° and 36.16°, respectively. In the crystal wrap, molecules are linked via two classical intermolecular hydrogen bonds between N1—H1N1···O1i and N3—H1N3···N2ii, Symmetry codes: (i) *x*, −*y*+1, *z*+1/2; and (ii) −*x*+1, *y*, −*z*+3/2 (Table S5).

### 2.3. Computational Studies

Comparison of the theoretical values with the experimental ones indicates that all the optimized bond lengths are closer to the experimental values. In the case of X-ray structure of compound **L1**, the observed bond lengths of C1–C10, O3–C15 and N3–N4 bonds in five-membered pyrazole ring are 1.227(3) Å, 1.363(3) Å and 1.383(3) Å, respectively. The calculated bond lengths, through DFT method, of same pyrazole ring are 1.24394 Å, 1.38125 Å and 1.37144 Å, respectively, which are very close to the actual values. In Table 1, it is clear that actual C-C and C-H bond lengths are also in close agreement with calculated values. The calculated bond angles for O1–C10–C9, C14–O2–C18 and N4–C11–C12 bond angles of **L1** are 122.22°, 118.52°, 121.37°, respectively, which are close to the corresponding actual angles obtained from X-ray. The actual values of above bond angles are 121.3°, 118.3°, and 122.9°, respectively.

**Figure 3:**
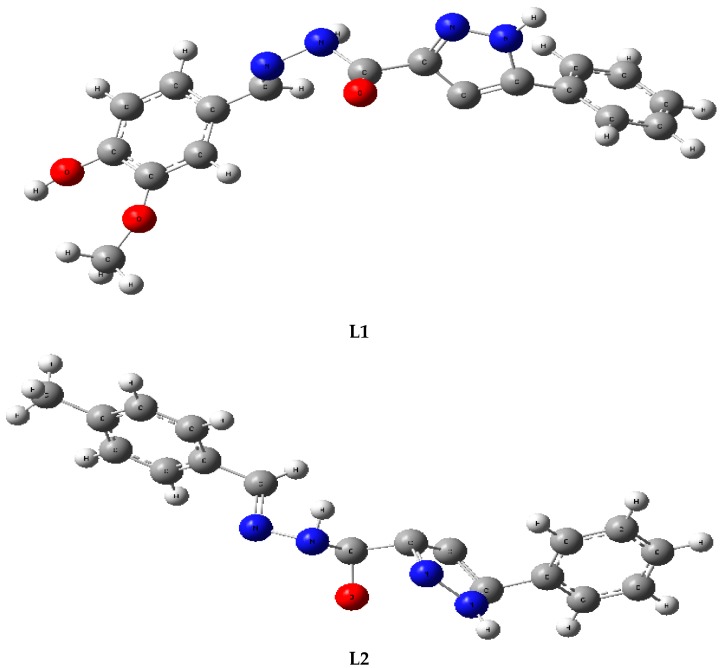
Optimized geometry of **L1** and **L2**.

The optimized geometry of compounds **L1** and **L2** were obtained at B3LYP/6-31G* level. Some optimized geometric parameters are also listed in Figure 3, and Table 2 and Table 3.

**Figure 4:**
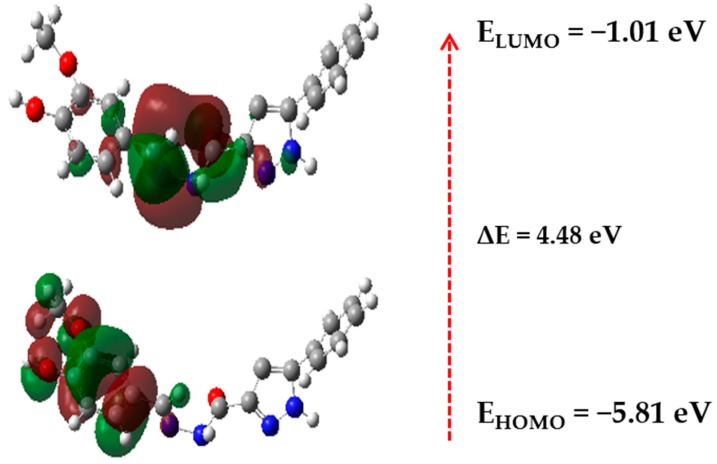
HOMO–LUMO energy diagram of **L1**.

**Figure 5:**
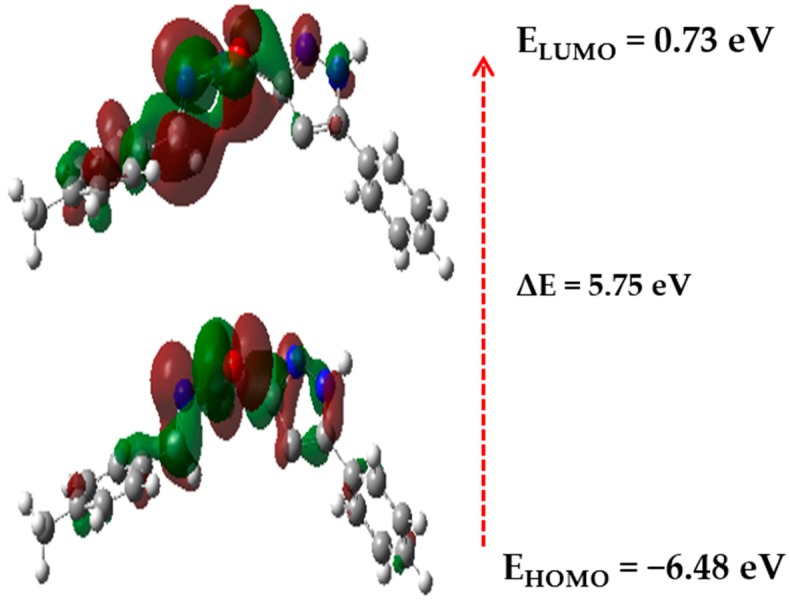
HOMO–LUMO energy diagram of **L2**.

The total energy, energy of HOMO and energy of LUMO, as well as other parameters for structures **L1** and **L2** are obtained theoretically and listed in Table 3. The HOMO and LUMO electron density distributions of **L1** and **L2** are given in Figure 4 and Figure 5, respectively. After the analysis of the theoretical results obtained, we can say that molecules **L1** and **L2** have a non-planar structure. DFT calculation gives an idea about the substance reactivity and site selectivity of the frameworks. EHOMO; ELUMO, which clarifies the inevitable charge exchange collaboration inside the studied material; electronegativity (χ); hardness (η); potential (μ); electrophilicity (ω); softness (S); and softness (*σ*) are recorded in Table 3. The significance of η and ϭ is to evaluate both the reactivity and stability.

The analysis of the wave function indicates that the energy space between the molecular orbit HOMO and LUMO determines the chemical stability and the electrical transport properties of the molecule. The red and green colors of the molecular orbital ridge, respectively, represent the positive and negative phases.

The HOMO of **L1** shows the charge density localized on the 4-hydroxy-3-methoxybenzaldehyde ring, while LUMO is characterized by a charge distribution on the hydrazone function, indicating that this moiety can influence the electron transition. The HOMO of **L2** has a localized charge density on the pyrazole and hydrazone function, but LUMO is characterized by a charge distribution on the 4-methylbenzaldehyde ring and the hydrazone function. The energy difference between HOMO and LUMO of **L1** and **L2** is about 4.38 and 5.75 eV, respectively.

The energy of the smaller band space increases the stability of the molecule. The molecular boundary orbitals of **L1** and **L2** (HOMO–LUMO) are shown in Figure 4 and Figure 5, respectively.

### 2.4. Catecholase Activity: Spectrophotometric Study

**Scheme 2:**
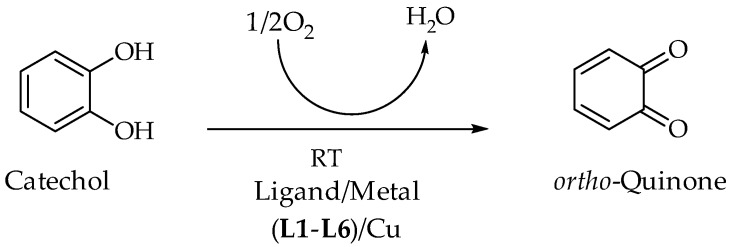
Catecholase reaction.

Catechol oxidation reaction catalyzed by copper complexes with ligands **L1**–**L6** is followed by the evolution of *o*-quinone absorbance measured at 390 nm with a UV-vis spectrometer (Scheme 2).

The catalytic oxidation rate variations from one complex to another are shown in Figures S1–S5. On the other hand, catechol oxidation rates were calculated and collected in Table 4. According to these results, we find that all copper complexes, formed in situ from ligands **L1**–**L6** and salts of copper, catalyze the oxidation reaction of catechol to *o*-quinone, but with different rates. The catalytic activities depend highly on the ligand concentration, the nature of solvent and the type of inorganic anion (Figures S1–S5).

It appears plainly that catalytic activity varies from one complex to another. All of the copper complexes formed by the hydrazone derivatives catalyze the oxidation reaction of catechol to *o*-quinone. The best is the **L6** ligand with highest oxidation rate for all copper salts with maximum reaction rate equal to 72.92 µmol·L^–1^·min^–1^ for CuSO_4_ followed by **L3** with maximum rate of 37.89 µmol·L^–1^·min^–1^ for Cu(NO_3_)_2_. Lowest recorded oxidation rates, whose values are less than 10 µmol·L^–1^·min^–1^, correspond to complex **L2**-CuCl_2_, followed by **L5**-(CuNO_3_)_2_ and **L4**-CuSO_4_, and then **L5**-CuCl_2_. The **L1** ligand seems to have nearly the same activity for all copper salts determined at about 10 µmol·L^–1^·min^–1^.

**Figure 6:**
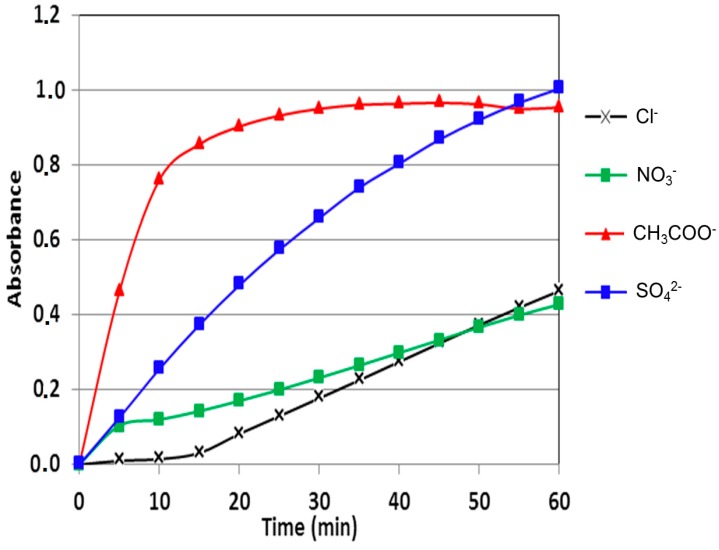
Catechol oxidation in the presence of copper complexes formed with **L6**.

Basically, for any ligand used except **L6**, Cu(CH_3_COO)_2_ gives highest activities and CuCl_2_ gives lowest activities (Figure 6). Concerning CH_3_COO^−^ and ligands **L3**, **L4** and **L6**, the values of absorbance increase at the beginning of the reaction and then remain constant after a certain time. This has been explained in previous studies by a precipitation of the complex that can take place during this decrease in absorbance.

#### 2.4.1. Effect of Ligand Concentration on the Catecholase Activity

**Figure 7:**
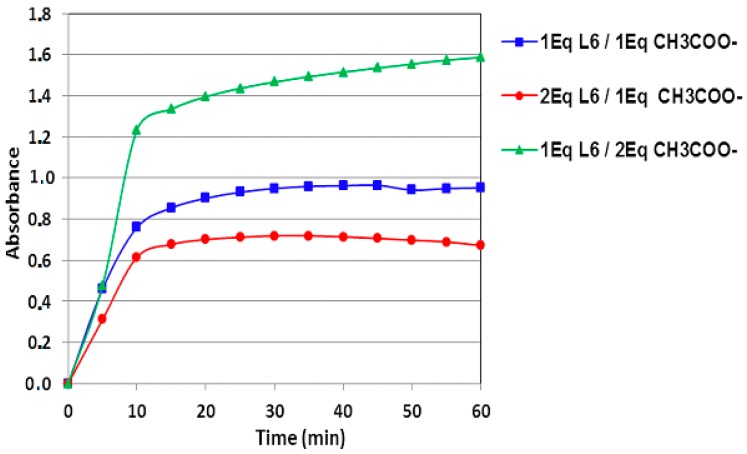
Catechol oxidation in methanol, in presence of formed **L6** copper complexes with different concentrations.

The effect of ligand concentration on catecholase activity is studied by varying the ratio of equivalent ligand **L6**: metallic salt Cu(CH_3_COO)_2_. Three tests were carried out in ratios: 1:1, 2:1 and 1:2. The results obtained show that the test with the ratio **L6**: Cu(CH_3_COO)_2_ = 1:2 leads to higher oxidation rate value. Therefore, the **L6** complex that contains two cupric ion Cu(II) has a better catalytic activity. However, ratios 2:1 and 1:1 lead to lower oxidation rate value, and ratio 2:1 exhibits maximum at 35 min, and after slightly decreases, probably due to complex precipitation (Figure 7).

#### 2.4.2. Solvent Effect

**Figure 8:**
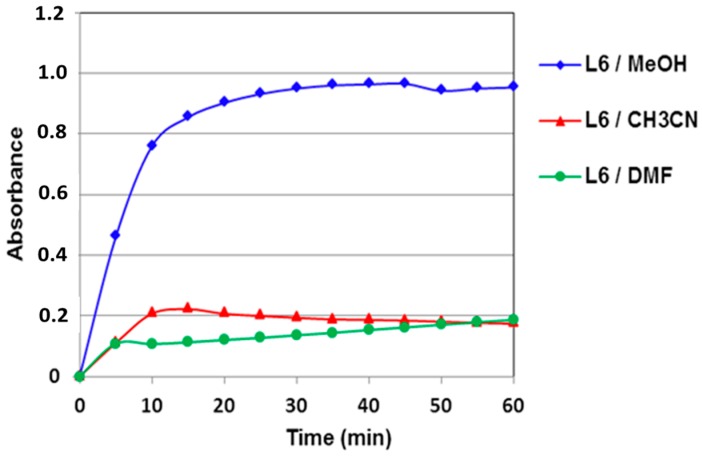
Catechol oxidation in different solvents and in the presence of formed **L6** copper complexes (1 Equivalent of **L_6_** for 1 Equivalent of Cu(CH_3_COOH)_2_).

The effect of three solvents (MeOH, CH_3_CN and DMF) on the oxidation reaction with the ligand **L6** and the Cu(CH_3_COO)_2_ salt is studied in similar thermodynamic conditions. The results of that study are presented by the rate values obtained for the oxidation reaction (Figure 8). Using a polar protic solvent such as methanol promotes oxidation reaction much better than the other two aprotic solvents, i.e., acetonitrile and DMF. Our results seem to be in perfect agreement with previous studies and show that solvatation of copper by the aprotic and polar solvents such as DMF and CH_3_CN decreases the catalytic activity of the metal cation in the oxidation reaction of catechol to *o*-quinone.

#### 2.4.3. Comparison with Alternative Catalysts

Table 5 shows the catalytic activity by other catalysts reported in the literature. It is clear that the hydrazone–pyrazole derivatives, in particular ligand **L6**, described in this work present further improvement and show better values and higher activity for the effective aerobic oxidation of the catechol into *o*-quinone.

The superior catalytic activity observed for ligand **L6** is probably due to the stability of corresponding copper complex (catalyst) favored by the organic conjugate π bonds of the three benzene ring contained in the ligand and by the intense coordination bonds of the Schiff base. To our knowledge, the catalytic activity observed for ligand **L6** (72.920 µmol·L^–1^·min^–1^) is the most important among the catalysts described in the literature.

## 3. Experimental

### 3.1. General Methods

The chemical reagents used in synthesis were purchased from Merck, Darmstadt, Germany or Aldrich, St. Louis, MO, USA; were of the highest commercially available purity; and were used without previous purification. Melting points were measured using a Büchi B-545 digital capillary melting point apparatus (BUCHI Labortechnik AG, Flawil, CH, Switzerland) and used without correction. Reactions were checked with TLC silica gel 60 F254 (MACHEREY-NAGEL, Neumann-Neander, Drun, Germany). Spectra IR were recorded on a VERTEX 70 FT-IR spectrometer (Perkin-Elmer, Billerica, MA, USA) and frequencies are reported in cm^−1^. The spectra of ^1^H NMR and ^13^C NMR were recorded in solution in DMSO-*d*_6_ on a Bruker Avance 300 NMR spectrometer (Bruker, MA, USA). The chemical shifts are expressed in parts per million (ppm) by using tetramethylsilane (TMS) as internal reference and coupling constants (J) are given in Hz. Mass spectra were collected using a API 3200 LC/MS/MS system (AB MDS Sciex, Ontario, Canada) equipped with an ESI source. DRX data were collected on a Bruker APEX-II D8 Venture area diffractometer (Bruker, MA, USA) equipped with graphite monochromatic Mo Kα radiation, λ = 0.71073 Å at 293 (2) and 296 (2) K, respectively. The electronic spectra of the ligand and its metal complexes ere measured on a Lambda 35 ES UV/VIS spectrophotometer (Perkin Elmer, Waltham, MA, USA) in the range of 200–900 nm.

### 3.2. Synthesis

#### 3.2.1. Synthesis of 3-phenyl-1*H*-pyrazole-4-carbohydrazides (2)

To a stirred solution of 1 mmol of the 3-phenyl-1*H*-pyrazole-4-carboxylate (**1**) in ethanol (10 mL), 2 mL of 80% hydrazine monohydrate was added. The reaction mixture was maintained under reflux for 5 h, until TLC indicated the end of reaction. Afterwards, the reaction mixture was poured onto ice and the solid formed was collected by filtration, washed with cold water and recrystallized from ethanol. Yield: 66%; m.p: 207–209 °C; IR (ν(cm^−1^)) : 3296–3203 (NH, NH_2_), 1629 (C=O); ^1^H NMR: (300 MHz, DMSO-*d*_6_, δ(ppm)) : 4.45 (s, 2*H*, NH_2_), 7.05 (1*H*, s, CH-pyrazole), 7.17–7.60 (5*H*, m, Ar-H), 9.38 (s, 1*H*, NHCO), 13.61 (1*H*, s, NH-pyrazole); ESI-MS: *m*/*z* = 203.3 [M+H]^+^, 225.1 [M+Na]^+^. The FT-IR, ^1^H NMR and mass spectra of compound **2** are shown in Figures S6–S8.

#### 3.2.2. General Procedure for the Synthesis of Ligands (**L1**–**L6**)

To a solution of 5-phenyl-*1H*-pyrazole-4-carbohydrazide (**2**) (1 mmol) in 10 mL of ethanol, an equimolar amount of the appropriate benzaldehyde derivative was added in the presence of acetic acid. The mixture was maintained under reflux for 2–5 h, until TLC indicated the end of reaction. Then, the reaction mixture was cooled to 25 °C, and the precipitate formed was filtered out washed with ethanol and recrystallized from ethanol. The FT-IR, (^1^H, ^13^C) NMR and mass spectra of Ligands (**L1**–**L6**) are shown in Figures S9–S30.

#### 3.2.3. *N*’-(4-hydroxy-3-methoxybenzylidene)-5-phenyl-1*H*-pyrazole-3-carbohydrazide (**L1**)

Yield: 85%; m.p: 229–231 °C; IR (ν(cm^−1^)): 3276 (NH), 1681 (C=O), 1568 (C=N); ^1^H NMR: (300 MHz, DMSO-*d*_6_, δ(ppm)): 3.81 (3H, s, -OCH_3_), 6.83 (d, J = 8.1 Hz, 1*H*, H-Ar), 7.18 (1*H*, s, CH-pyrazole), 7.26–7.49 (5*H*, m, Ar-H), 7.54 (s, 1*H*, H-Ar), 7.82 (d, J = 8.1 Hz, 1*H*, H-Ar), 8.39 (1*H*, s, N=CH), 11.51 (s, 1*H*, OH), 11.72 (s, 1*H*, NHCO), 13.72 (1*H*, s, NH-pyrazole); ^13^C NMR: (300 MHz, DMSO-*d*_6_, δ (ppm)): 56.04 (OCH_3_), 103.85 (CH, C4-pyrazole), 115.91 (CH, C-Ar), 122.53 (CH, C-Ar), 125.53 (CH, C-Ar), 126.37 (CH, C-Ar), 128.28 (CH, C-Ar), 129.07 (CHC-Ar), 129.33 (C, C-Ar), 129.54 (C-Ar), 144.12 (CH, N=CH), 145.85 (C, C3-pyrazole), 147.36 (C, C5-pyrazole), 148.63 (C, C-OH), 156.48 (C, C-OCH_3_), 158.40 (C, C=O). ESI-MS: *m*/*z* = 337.0 [M+H]^+^, 359.0 [M+Na]^+^.

#### 3.2.4. *N*’-(4-methylbenzylidene)-5-phenyl-1*H*-pyrazole-3-carbohydrazide (**L2**)

Yield: 90%; m.p: 297–299 °C; IR (ν(cm^−1^)): 3205 (NH), 1680 (C=O),1561 (C=N); ^1^H NMR: (300 MHz, DMSO-*d*_6_, δ(ppm)): 2.32 (3*H*, s, CH_3_), 7.25 (1*H*, s, CH-pyrazole), 7.33–7.61 (5*H*, m, Ar-H), 7.59 (d, J = 7.8 Hz, 2*H*, H-Ar), 7.81 (d, J = 7.8 Hz, 1*H*, H-Ar), 8.45 (1*H*, s, N=CH), 11.65 (s, 1*H*, NHCO), 13.78 (1*H*, s, NH-pyrazole); ^13^C NMR: (300 MHz, DMSO-*d*_6_, δ (ppm)): 21.50 (OCH_3_), 103.85 (CH, C4-pyrazole), 125.80 (CH, C-Ar), 127.53 (CH, C-Ar), 127.90 (CH, C-Ar), 128.94 (CH, C-Ar), 129.49 (CH, C-Ar), 129.92 (C, C-Ar), 132.18 (C, C-Ar), 136.80 (C, C-CH_3_), 140.28 (C, C3-pyrazole), 146.18 (CH, N=CH), 148.28 (C, C5-pyrazole), 158.50 (C, C=O). ESI-MS: *m*/*z* = 304.9 [M+H]^+^, 326.9 [M+Na]^+^.

#### 3.2.5. *N*’-(4-chlorobenzylidene)-5-phenyl-1*H*-pyrazole-3-carbohydrazide (**L3**)

Yield: 89%; m.p: 301–303 °C; IR (ν(cm^−1^)): 3207 (NH), 1680 (C=O), 1605 (C=N); ^1^H NMR: (300 MHz, DMSO-*d*_6_, δ(ppm)): 7.11 (1*H*, s, CH-pyrazole), 7.33–7.60 (5*H*, m, Ar-H), 7.59 (d, J = 7.2 Hz, 2*H*, H-Ar), 7.81 (d, J = 7.2 Hz, 1*H*, H-Ar), 8.46 (1*H*, s, N=CH), 11.65 (s, 1*H*, NHCO), 13.79 (1*H*, s, NH-pyrazole); ^13^C NMR: (300 MHz, DMSO-*d*_6_, δ (ppm)): 103.82 (CH, C4-pyrazole), 125.82 (CH, C-Ar), 127.53 (CH, C-Ar), 127.90 (CH, C-Ar), 128.99 (CH, C-Ar), 129.49 (CH, C-Ar), 129.91 (C, C-Ar), 132.18 (C, C-Ar), 140.28 (C, C- CH_3_), 148.20 (C, C3-pyrazole), 156.88 (CH, N=CH), 158.62 (C, C5-pyrazole), 164.13 (C, C=O). ESI-MS: *m*/*z* = 325.1 [M+H]^+^, 347.3 [M+Na]^+^.

#### 3.2.6. *N*’-(4-fluorobenzylidene)-5-phenyl-1*H*-pyrazole-3-carbohydrazide (**L4**)

Yield: 98%; m.p: 294–296 °C; IR (ν(cm^−1^)) : 3320 (NH), 1672 (C=O), 1604 (C=N); ^1^H NMR: (300 MHz, DMSO-*d*_6_, δ(ppm)): 7.21 (1*H*, s, CH-pyrazole), 7.25–7.38 (5*H*, m, Ar-H), 7.42 (d, J = 7.8 Hz, 2*H*, H-Ar), 7.81 (d, J = 7.8 Hz, 1*H*, H-Ar), 8.50 (1*H*, s, N=CH), 11.72 (s, 1*H*, NHCO), 13.79 (1*H*, s, NH-pyrazole); ^13^C NMR: (300 MHz, DMSO-*d*_6_, δ (ppm)): 103.86 (CH, C4-pyrazole), 125.82 (CH, CH-Ar), 128.98 (CH, C-Ar), 129.49 (CH, CH-Ar), 129.64 (C, C-Ar), 129.74 (CH, CH-Ar), 130.10 (C, CH-Ar), 130.22 (C, C-Ar), 146.97 (C, C3-pyrazole), 150.50 (CH, N=CH), 156.68 (C, C5-pyrazole), 161.90 (C, C=O), 165.18 (C, C-F). ESI-MS: *m*/*z* = 309.3 [M+H]^+^.

#### 3.2.7. 5-phenyl-*N*’-(1-phenylethylidene)-1*H*-pyrazole-3-carbohydrazide (**L5**)

Yield: 85%; m.p: 251–253 °C; IR (ν(cm^−1^)) : 3320 (NH), 1667 (C=O),1589 (C=N); ^1^H NMR: (300 MHz, DMSO-*d*_6_, δ(ppm)): 2.36 (3*H*, s, CH_3_), 7.24 (1*H*, s, CH-pyrazole), 7.42–7.84 (10*H*, m, Ar-H), 10.37 (s, 1*H*, NHCO), 13.82 (1*H*, s, NH-pyrazole); ^13^C NMR: (300 MHz, DMSO-*d*_6_, δ (ppm)): 21.51 (OCH_3_), 103.60 (CH, C4-pyrazole), 125.73 (CH, C-Ar), 127.72 (CH, C-Ar), 128.70 (CH, C-Ar), 129.01 (CH, C-Ar), 129.18 (CH, C-Ar), 129.56 (CH, C-Ar), 130.34 (C, C-Ar), 132.07 (C, C-Ar), 137.21 (C, C3-pyrazole), 144.82 (CH, N=CH), 146.45 (C, C5-pyrazole), 157.23 (C, C=O). ESI-MS: *m*/*z* = 305.4 [M+H]^+^.

#### 3.2.8. *N*’-(diphenylmethylene)-5-phenyl-1*H*-pyrazole-3-carbohydrazide (**L6**)

Yield: 82%; m.p: 200–202 °C; IR (ν(cm^−1^)): 3360 (NH), 1664 (C=O), 1537 (C=N); ^1^H NMR: (300 MHz, DMSO-*d*_6_, δ(ppm)): 7.20 (1*H*, s, CH-pyrazole), 7.32–7.76 (15*H*, m, Ar-H), 9.89 (s, 1*H*, NHCO), 13.84 (1*H*, s, NH-pyrazole); ^13^C NMR: (300 MHz, DMSO-*d*_6_, δ (ppm)): 105.10 (CH, C4-pyrazole), 127.66 (CH, C-Ar), 128.66 (CH, C-Ar), 128.96 (CH, C-Ar), 130.21 (CH, C-Ar), 130.31 (CH, C-Ar), 130.46 (CH, C-Ar), 132.02 (C, C-Ar), 137.28 (C, C-Ar), 141.08 (C, C3-pyrazole), 145.70 (CH, N=CH), 153.35 (C, C5-pyrazole), 157.62 (C, C=O). ESI-MS: *m*/*z* = 366.9 [M+H]^+^, 389.0 [M+H]^+^.

### 3.3. X-Ray Crystallographic Analysis

The compounds of **L1** and **L2** were obtained as single crystals by slow evaporation from ethanol solution of the pure compound at room temperature. Cell refinement and data reduction were carried out by Bruker SAINT. SHELXT was used to solve structure [36,37]. The final refinement was carried out by full-matrix least-squares techniques with anisotropic thermal data for no hydrogen atoms on 𝐹. CCDC 1522882 and 1523265 for **L1** and **L2**, respectively, contain the supplementary crystallographic data for these compounds can be obtained free of charge from the Cambridge Crystallographic Data Centre via http://www.ccdc.cam.ac.uk/data_request/cif.

### 3.4. DFT Computational Method

The computational studies of compounds **L1** and **L2** were performed at the B3LYP/6-31G level of theory using Gaussian 09 package programs [38,39]. The optimization geometries of **L1** and **L2** were performed using the Berny analytical gradient optimization method [40].

### 3.5. Catecholase Activity Measurement

Kinetic measurements were made spectrophotometrically on UV-vis spectrometer, following the appearance of *o*-quinone over time at 25 °C (390 nm absorbance maximum *ε* = 1600 L mol^−1^ cm^−1^ in methanol [35]. The complexes were prepared in situ by successively mixing 0.15 mL of a solution (2 × 10^−3^ M) of CuX_2_·nH_2_O (X = Cl^−^, NO_3_^−^, CH_3_COO^−^ or SO_4_^2−^), with 0.15 mL of a solution (2 × 10^−3^ M) of ligand, then adding 2 mL of a solution of catechol at a concentration of 10^−1^ M.

## 4. Conclusions

In this work, we report the synthesis of six new hydrazone–pyrazole biomolecules (**L1**–**L6**) in excellent yields. The X-ray structures of **L1** and **L2** have been investigated herein for the first time. The theoretical calculations through DFT of **L1** and **L2** well supported the experimental findings. These ligands (**L1**–**L6**) and different Cu(II) salts demonstrate an efficient activity to catalyze the aerobic oxidation of the catechol into *o*-quinone compared to others recent catalysts described in the literatures. Interestingly, ligand **L6** exhibits an extremely high rate of oxidation, attaining 72.92 μmol·L^−1^·min^−1^, which is, to our knowledge, the best catalytic activity among the reported catalysts. Cu(II)-ligand complexes were generated in situ and the results obtained show that the oxidation depend highly on several parameters: the nature and concentration of the ligand, the nature of salts and the solvent effects. The results suggest that these new materials have potential for the oidation of the catechol into *o*-quinone, thus opening important perspectives.

## Figures and Tables

**Table 1:** Selected structural parameters by X-ray and theoretical calculations of compound **L_1_**.

Bond Length (Å)	Experimental Bond Lengths	Calculated Bond Lengths	Bond Angle (°)	Experimental Bond Angles	Calculated Bond Angles
O1–C10	1.22(3)	1.24	N2–C9–C8	106.0(2)	109.2
O2–C14	1.36(3)	1.39	O1–C10–N3	122.8(2)	125.0
O2–C18	1.41(4)	1.45	N3–C10–C9	116.0(2)	112.7
O3–C15	1.36(3)	1.38	O1–C10–C9	121.3(2)	122.2
N1–N2	1.33(3)	1.37	N4–C11–C12	122.9(2)	121.3
N1–C7	1.33(3)	1.38	O2–C14–C13	125.7(2)	126.0
N2–C9	1.34(3)	1.36	C14–O2–C18	118.3(2)	118.5
N3–N4	1.38(3)	1.37	N2–N1–C7	105.3(2)	113.2
N3–C10	1.34(3)	1.38	N1–N2–C9	112.6(2)	105.0
N4–C11	1.27(3)	1.29	N4–N3–C10	119.5(2)	121.0

**Table 2:** Selected structural parameters by X-ray and theoretical calculations of compound **L2**.

Bond Length (Å)	Experimental Bond Lengths	Calculated Bond Lengths	Bond Angle (°)	Experimental Bond Angles	Calculated Bond Angles
O1–C10	1.22(2)	1.21	N2–N1–C7	113.4(15)	113.6
N1–N2	1.34(2)	1.34	N1–N2–C9	103.9(15)	105.4
N1–C7	1.34(3)	1.37	N4–N3–C10	119.2(17)	117.2
N2–C9	1.33(2)	1.34	N3–N4–C11	115.6(18)	114.2
N3–N4	1.38(2)	1.41	N1–C7–C6	122.7(17)	122.39
N3–C10	1.33(3)	1.36	N1–C7–C8	105.3(18)	103.59
N4–C11	1.26(3)	1.27	N2–C9–C8	111.4(16)	109.21
C15–C18	1.512(4)	1.50	N2–C9–C10	119.4(17)	119.83
N1–H	0.86(2)	1.00	N3–C10–C9	114.9(16)	111.05

**Table 3:** Calculated energies of **L1** and **L2**.

Molecular Energy (a.u.) (eV)	L1	L2
*TE*	−31008.6	−26911.4
E_HOMO_	−5.8186	−6.4850
E_LUMO_	−1.0152	−0.7349
Gap *ΔE*	4.8034	5.7500
Chemical potential *µ* (D)	5.8871	6.3122
Ionization potential (IP)	5.8186	6.4850
Electron affinity (EA)	1.0152	0.7349
Electron negatiity (*χ*)	3.4169	3.6100
Global hardness (*η*)	2.4017	2.875
Global electrophilicity (*ω*)	2.4306	2.2664

**Table 4:** Oxidation rate of catechol oxidation in methanol (µmol·L^−1^·min^−1^).

Ligand/Metallic Salt	Cu(NO_3_)_2_	CuCl_2_	Cu(CH_3_COO)_2_	CuSO_4_
**L1**	10.57	10.28	9.80	9.27
**L2**	15.52	0.05	22.92	16.06
**L3**	37.89	9.19	24.58	21.01
**L4**	17.71	11.43	15.02	6.57
**L5**	3.10	8.07	19.62	10.61
**L6**	27.77	40.27	60.50	72.92

**Table 5:** Comparison of the catalytic activity of various catalysts toward oxidation of the catechol into *o*-quinone, established in the same conditions, as given in previous literature.

Cu(II)-Ligands	Cu(II) Salt Used	Oxidation Rate (µmol·L^−1^·min^−1^)	Ref.
ligand **L6**	CuSO_4_	**72.920**	-
ligand **L6**	Cu(CH_3_COO)_2_	**60.500**	-
ligand **L6**	CuCl_2_	**40.270**	-
*C*,*N*-bipyrazole	Cu(CH_3_COO)_2_	4.440	[29]
bipyrazolic tripode-prop-2-ylacetate	Cu(CH_3_COO)_2_	11.825	[30]
bipyrazolic tripode-4-hydroxyphenyl	CuCl_2_	1.458	[31]
bipyrazolic tripode-3-hydroxypropyl	CuSO_4_	28.990	[32]
bipyrazolic tripode-3-hydroxypropyl	CuCl_2_	4.378	[33]
indole-3-chalcone	Cu(CH_3_COO)_2_	31.780	[34]
[(3,5-dimethyl-pyrazol-1-ylmethyl)-amino]-propionitrile	CuSO_4_	8.710	[35]

## Supplementary Materials

Supplementary materials can be found at www.mdpi.com/1422-0067/18/11/2215/s1.
