# Supplementary material for: New Pyrazole-Hydrazone Derivatives: X-ray Analysis, Molecular Structure Investigation via Density Functional Theory (DFT) and Their High In-Situ Catecholase Activity
Source: Int J Mol Sci. 2017 Oct 25;18(11):2215. doi: 10.3390/ijms18112215 (PMC5713199; doi:10.3390/ijms18112215)
Supplement: Supplementary file 1 [file ijms-18-02215-s001.pdf]

Table S1. Refinement parameters and crystal data for **L1** and **L2.**

**CCDC Deposition Number**	**L1: 1522882**	**L2: 1523265**
Molecular Formula	$C_{18}H_{16}N_4O_3$	$C_{18}H_{16}N_4O$
Molecular Weight	336.12	304.13
Crystal System	Monoclinic	Monoclinic
Space Group	$P2_1/c$	C2/c
a (Å)	8.1405 (4)	18.2409 (13)
b (Å)	24.3478 (13)	14.7347 (13)
c (Å)	12.0975 (6)	13.1676 (9)
β (°)	114.087 (3)	106.072 (3)
V (Å^3)	2188.98 (19)	3400.8 (5)
Z	4	8
Dcalc (g·cm^{-3})	1.297	1.189
Crystal Dimension (mm)	0.36 × 0.28 × 0.14	0.42 × 0.22 × 0.07
μ (mm^{-1})	0.10	0.08
Tmin/Tmax	0.635/ 0.884	0.969/ 0.995
Measured Reflections	32643	61162
Indices Range (h, k, l)	−10/10, −31/31, −14/15	−23/23, −19/19, −17/17
θ Limit (°)	27.5-2.5	27.5-2.2
Unique Reflections	5028	3907
Observed Reflections (I > 2σ(I))	2561	2181
Parameters	308	217
Goodness of Fit on F^2	1.01	1.03
R_1,wR_2 [I > 2σ(I)]	0.0585/ 0.1548	0.0561/ 0.1514

Table S2. Selected geometric parameters (Å, °) for **L1**

O1—C10	1.227 (3)	N2—C9	1.346 (3)
O2—C14	1.363 (3)	N3—N4	1.383 (3)
O2—C18	1.417 (4)	N3—C10	1.341 (3)
O3—C15	1.363 (3)	N4—C11	1.274 (3)
O4—C21	1.200 (6)	N5—C21	1.313 (5)
N1—N2	1.339 (3)	N5—C19	1.444 (5)
N1—C7	1.334 (3)	N5—C20	1.445 (4)
C14—O2—C18	118.3 (2)	N2—C9—C8	106.0 (2)
N2—N1—C7	105.30 (19)	O1—C10—N3	122.8 (2)
N1—N2—C9	112.6 (2)	N3—C10—C9	116.0 (2)
N4—N3—C10	119.5 (2)	O1—C10—C9	121.3 (2)
N3—N4—C11	114.9 (2)	N4—C11—C12	122.9 (2)
C19—N5—C20	116.9 (3)	O2—C14—C13	125.7 (2)

C19—N5—C21	122.6 (3)	O2—C14—C15	114.2 (2)
C20—N5—C21	120.6 (3)	O3—C15—C14	117.2 (2)
N1—C7—C6	120.8 (2)	O3—C15—C16	123.4 (2)
N1—C7—C8	110.0 (2)	O4—C21—N5	126.3 (4)
N2—C9—C10	118.7 (2)		

Table S3 Hydrogen-bond geometry (Å, °) for **L1**

D—H···*A*	*D*—H	H···*A*	*D*···*A*	*D*—H···*A*
N3—H1N3···O3^{i}	0.87 (3)	2.24 (3)	3.067 (3)	160 (2)
O3—H1O3···O5W	0.95 (3)	1.71 (3)	2.652 (3)	171 (3)
N2—H1N2···O1^{ii}	0.90 (3)	1.98 (3)	2.811 (3)	154 (2)
O5W—H2OW···O1^{iii}	0.86 (4)	2.28 (3)	2.936 (3)	133 (3)
O5W—H2OW···N4^{iii}	0.86 (4)	2.37 (3)	3.160 (3)	153 (3)
O5W—H1OW···N1^{iv}	1.01 (4)	1.82 (4)	2.823 (3)	172 (3)
C8—H8A···O3^{i}	0.9300	2.5800	3.392 (3)	147.00
C13—H13A···O5W^{v}	0.9300	2.5500	3.463 (3)	168.00
C17—H17A···O4	0.9300	2.4300	3.316 (4)	158.00
Symmetry codes: (i) *x*+1, −*y*+1/2, *z*+1/2; (ii) −*x*, −*y*+1, −*z*+1; (iii) *x*, −*y*+1/2, *z*−1/2; (iv) −*x*, *y*−1/2, −*z*+1/2; (v) *x*, −*y*+1/2, *z*+1/2.				

Table S4 Selected geometric parameters (Å, °) for **L2**

O1—C10	1.226 (2)	N3—N4	1.382 (2)
N1—N2	1.340 (2)	N3—C10	1.338 (3)
N1—C7	1.349 (3)	N4—C11	1.266 (3)
N2—C9	1.335 (2)		
N2—N1—C7	113.43 (15)	N2—C9—C8	111.44 (16)
N1—N2—C9	103.94 (15)	N2—C9—C10	119.43 (17)
N4—N3—C10	119.23 (17)	N3—C10—C9	114.96 (16)
N3—N4—C11	115.62 (18)	O1—C10—N3	124.01 (19)
N1—C7—C6	122.70 (17)	O1—C10—C9	120.99 (19)

N1—C7—C8	105.38 (18)	N4—C11—C12	121.9 (2)

Table S5 Hydrogen-bond geometry (Å, °) For **L2**

D—H···*A*	*D*—H	H···*A*	*D*···*A*	*D*—H···*A*
N1—H1N1···O1^{i}	0.86 (2)	2.00 (2)	2.779 (2)	151 (2)
N3—H1N3···N2^{ii}	0.91 (2)	2.11 (2)	2.976 (2)	159.4 (19)
Symmetry codes: (i) *x*, −*y*+1, *z*+1/2; (ii) −*x*+1, *y*, −*z*+3/2.				

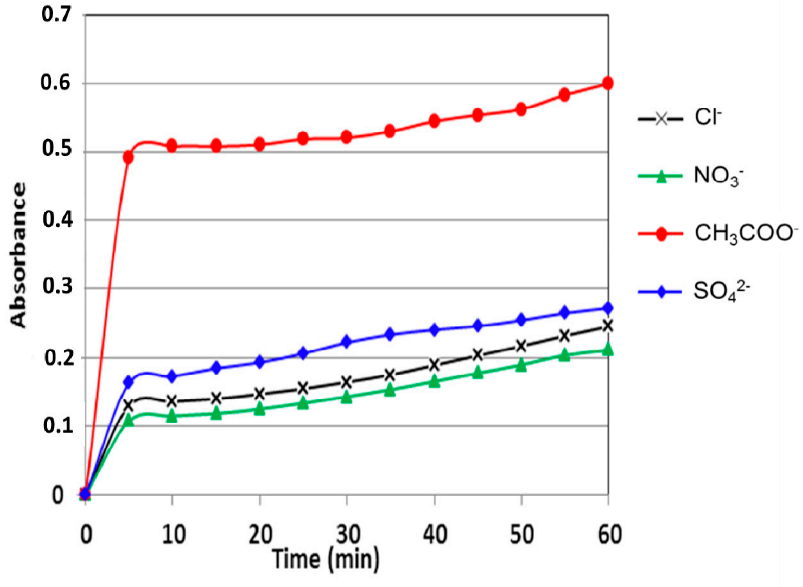

Figure S1. Catechol oxidation in the presence of copper complexes formed with **L1.**

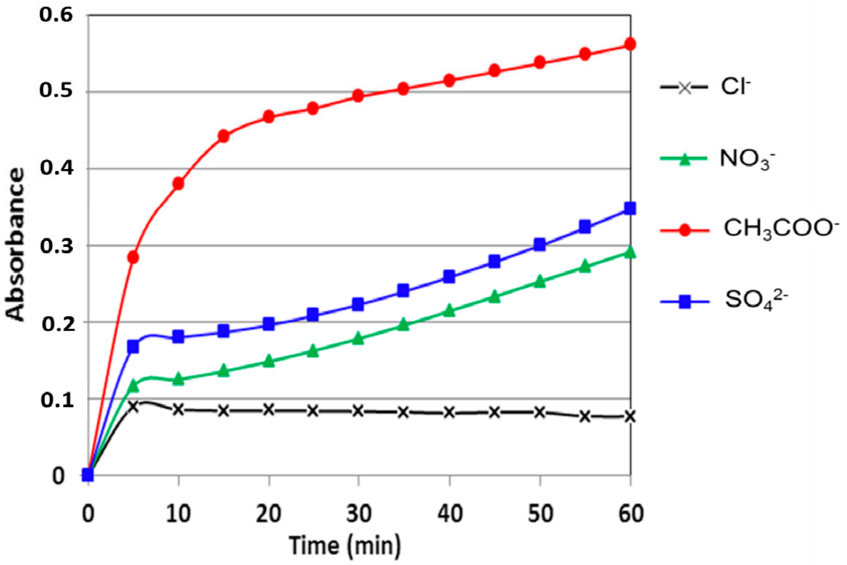

Figure S2. Catechol oxidation in the presence of copper complexes formed with **L2.**

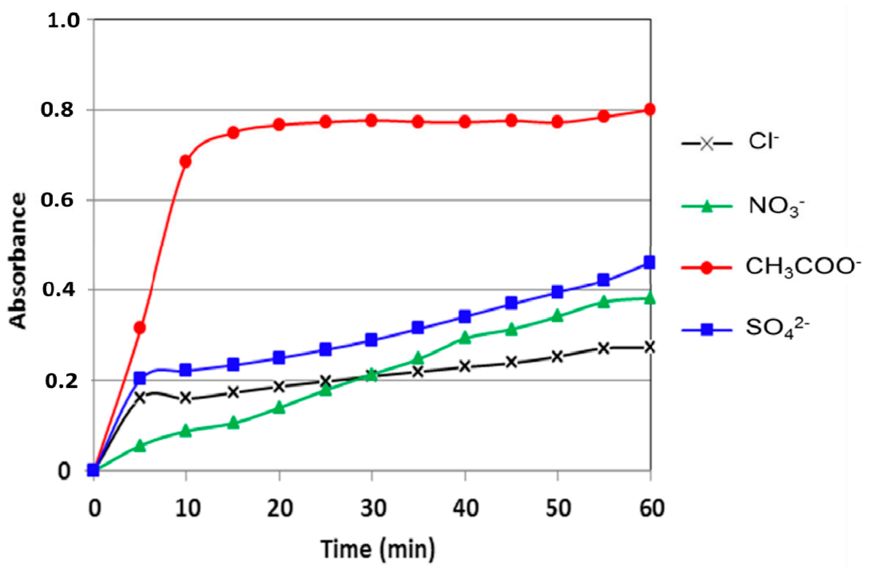

Figure S3. Catechol oxidation in the presence of copper complexes formed with $\mathbf{L_3}$.

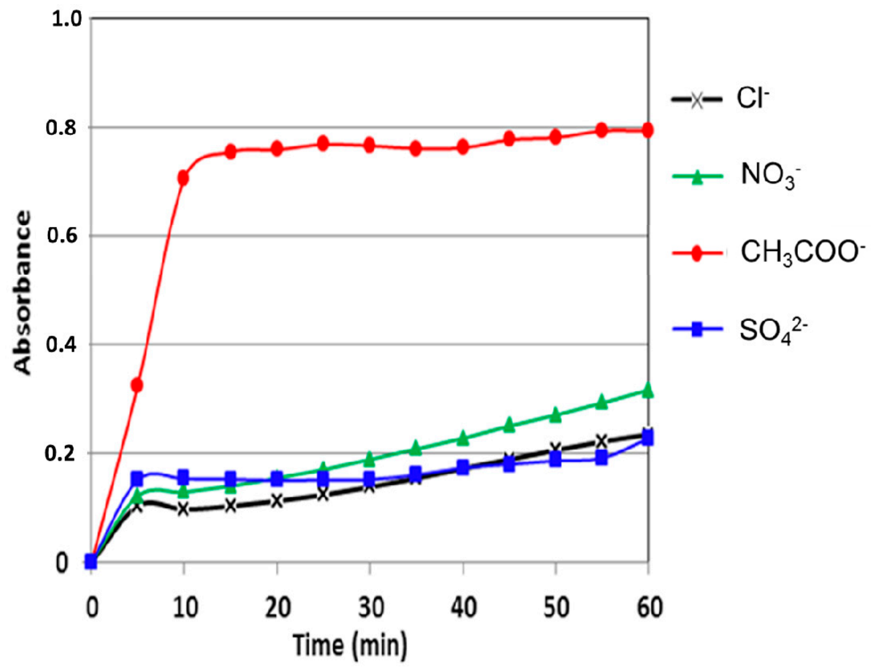

Figure S4. Catechol oxidation in the presence of copper complexes formed with $\mathbf{L_4}$.

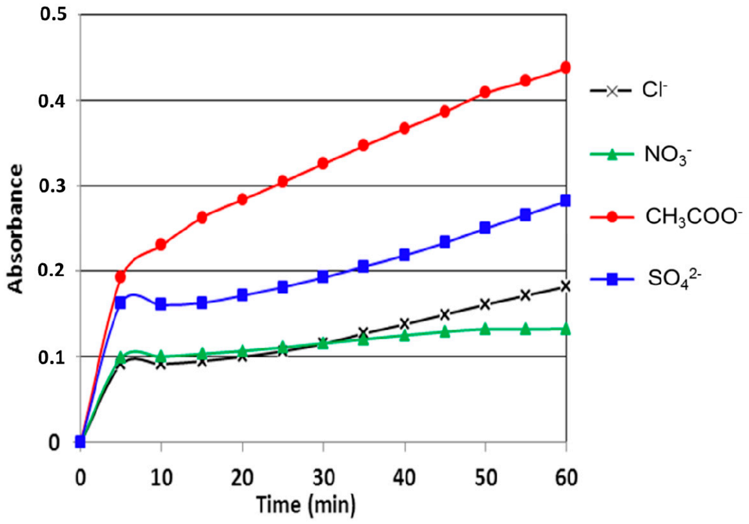

Figure S5. Catechol oxidation in the presence of copper complexes formed with $\mathbf{L_5}$.

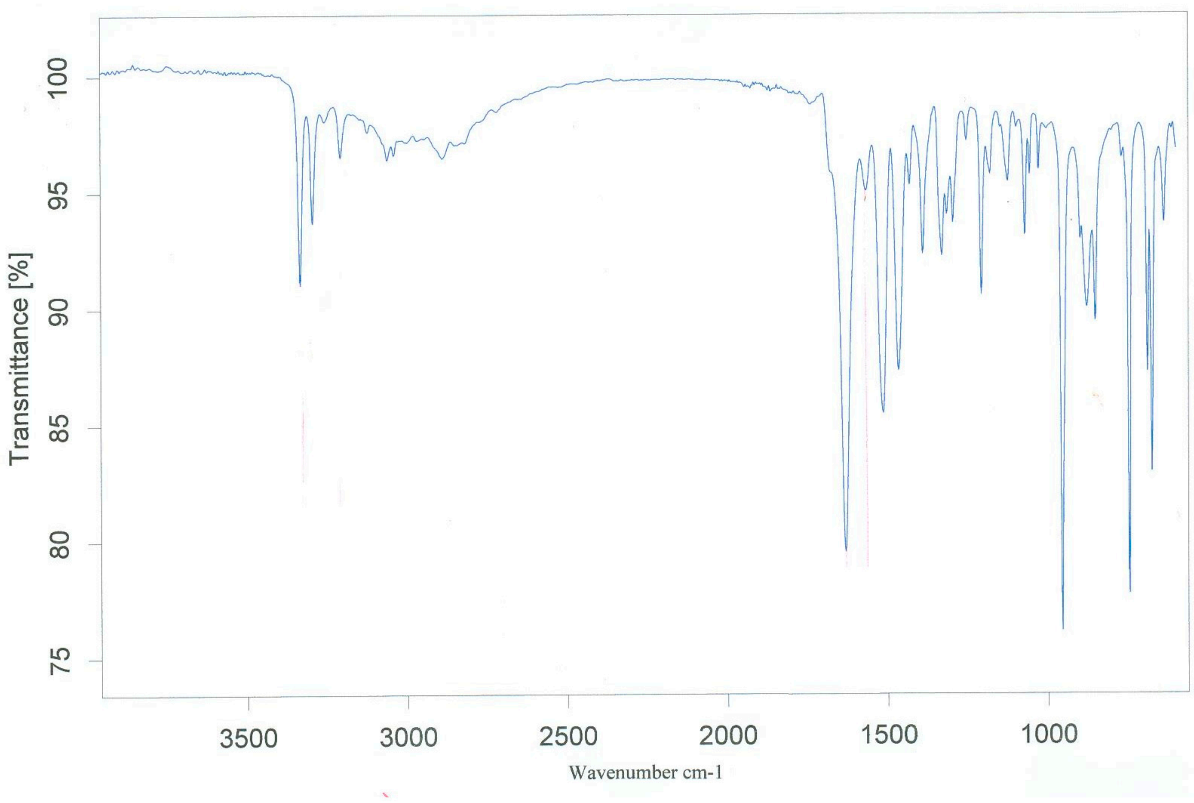

Figure S6. FT-IR spectrum of **2**.

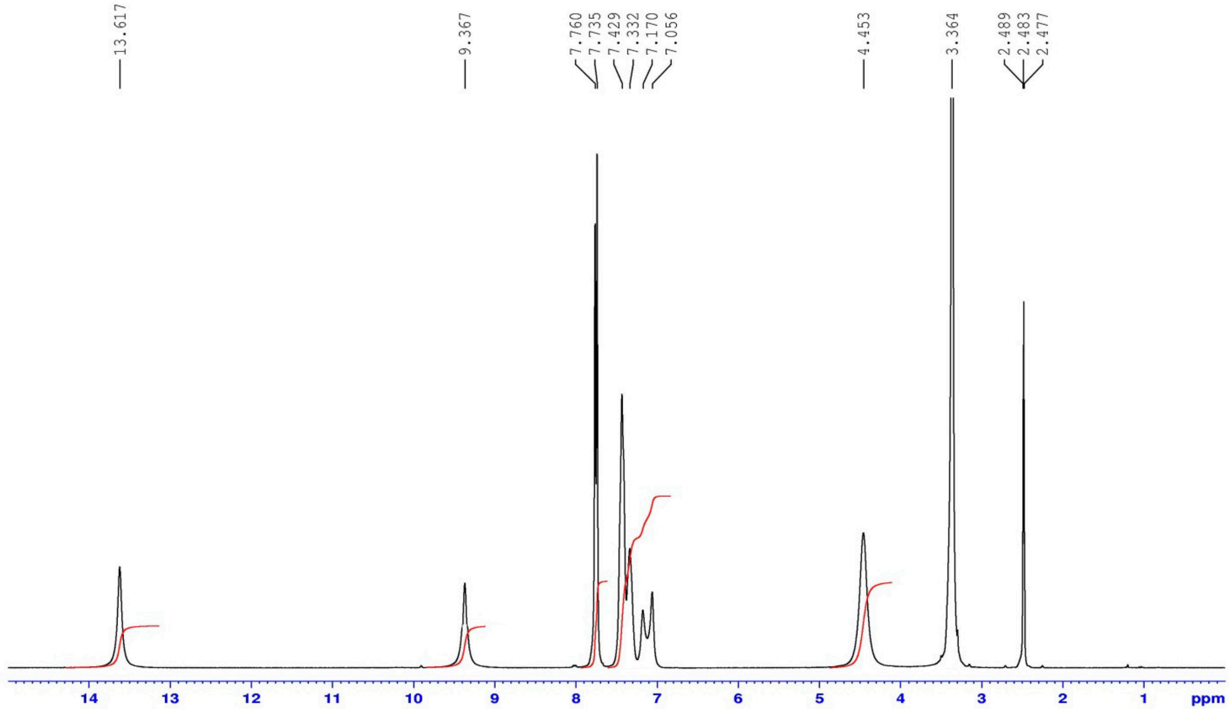

Figure S7. ^{1}H NMR spectrum of **2**.

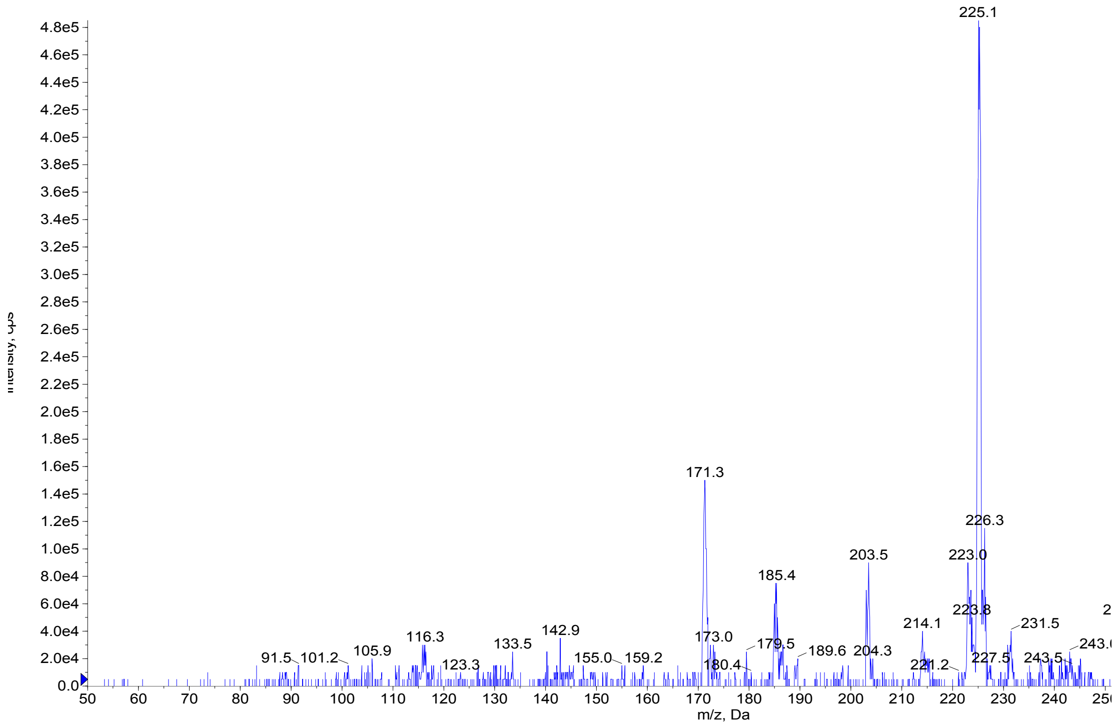

Figure S8. Mass spectrum of **2**.

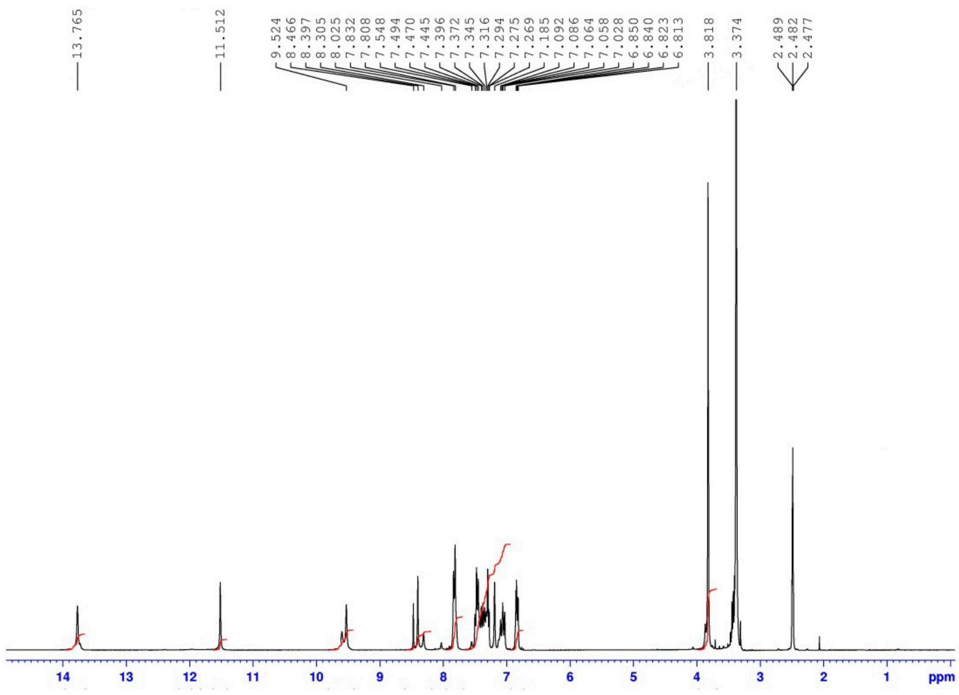

Figure S9. ^{1}H NMR spectrum of **L1**.

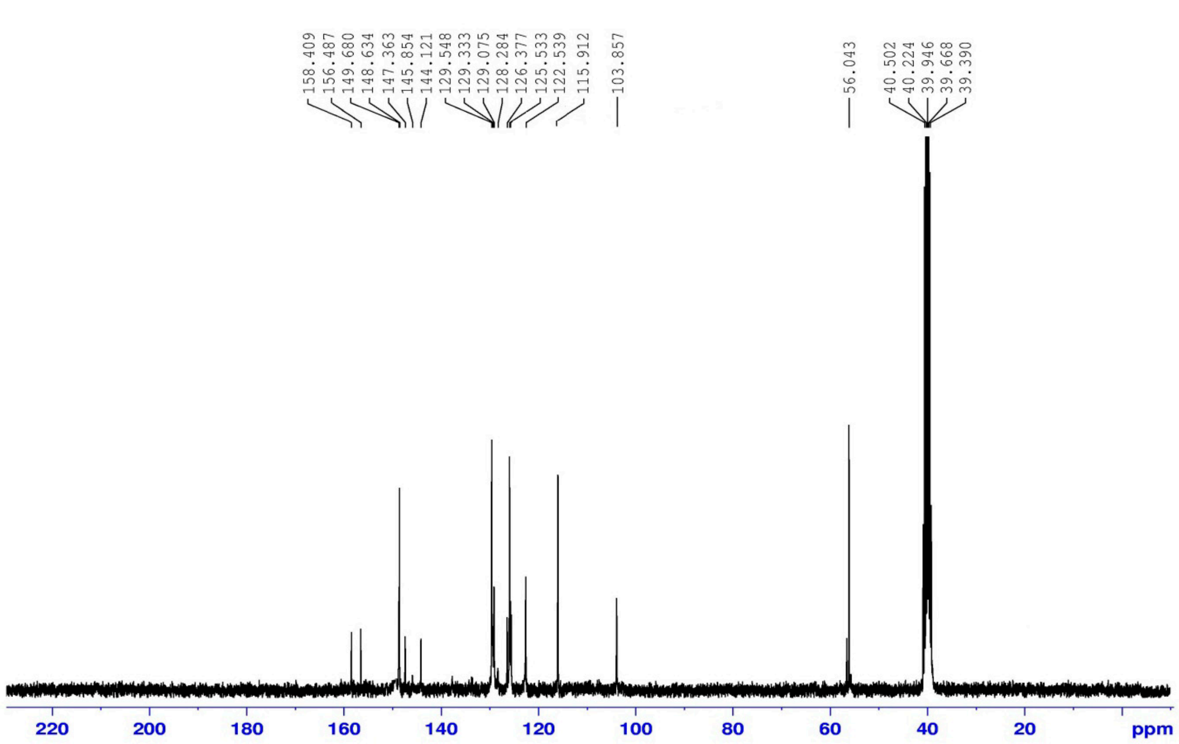

Figure S10. ^{13}C NMR spectrum of **L1**.

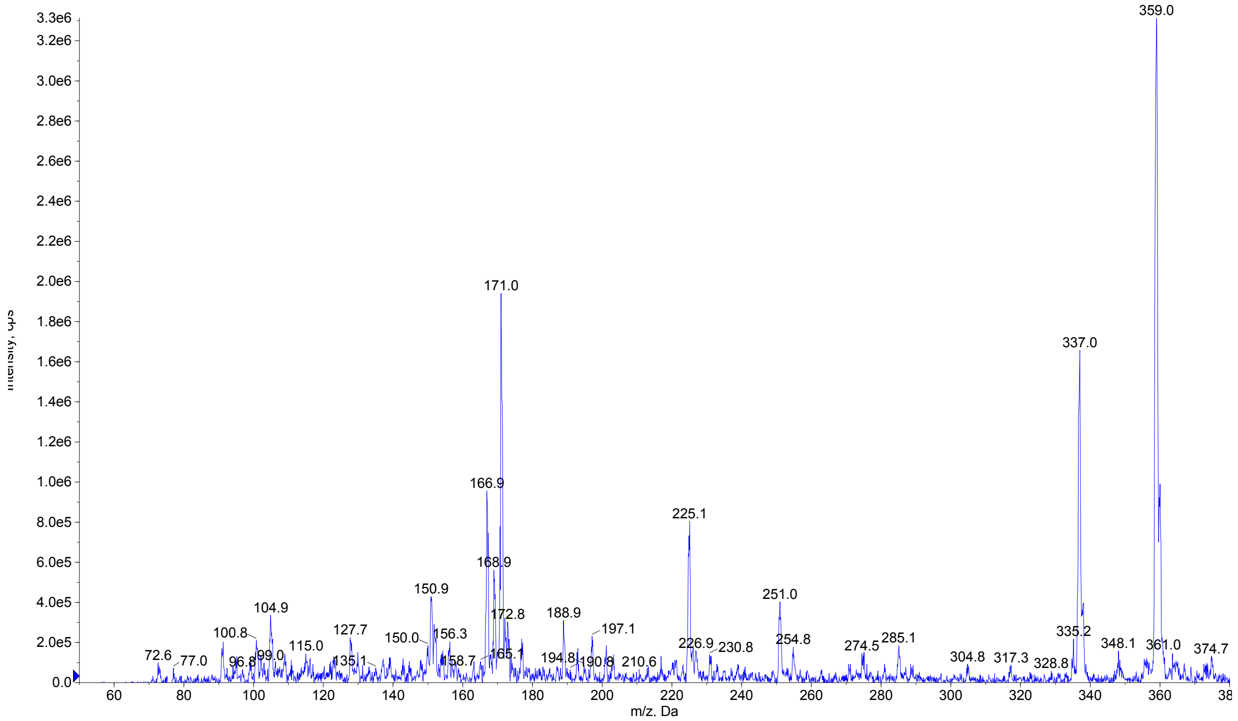

Figure S11. Mass spectrum of **L1**.

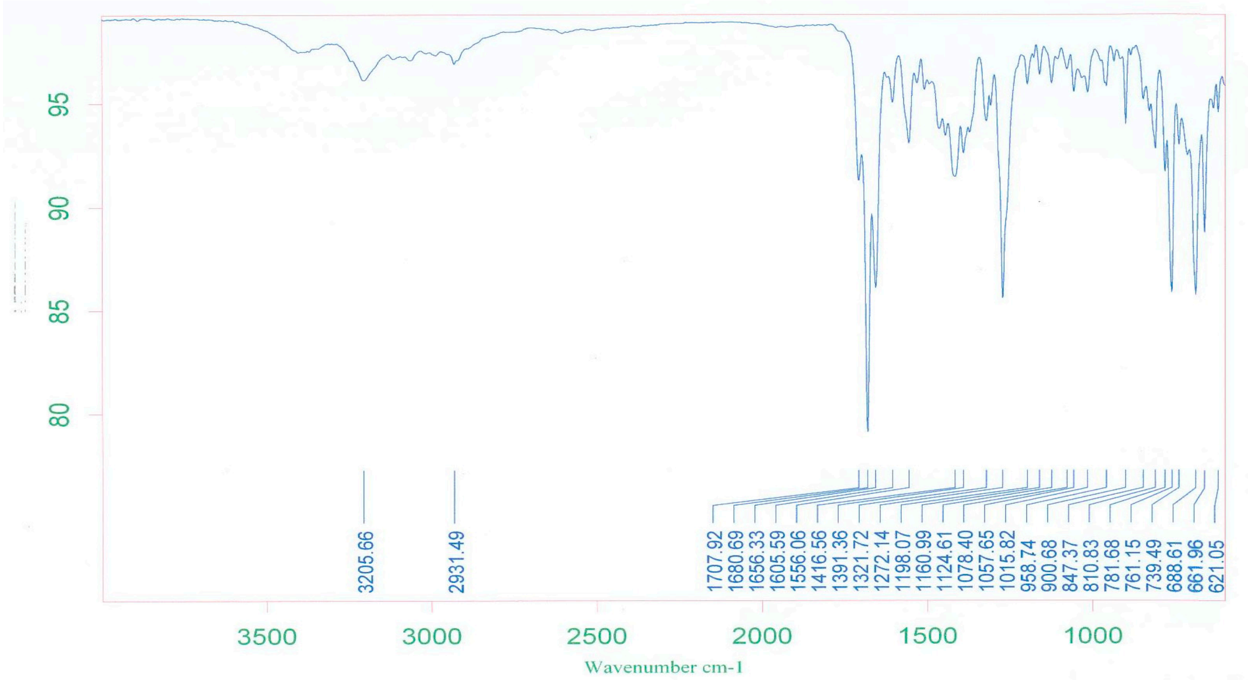

Figure S12. FT-IR spectrum of **L2**.

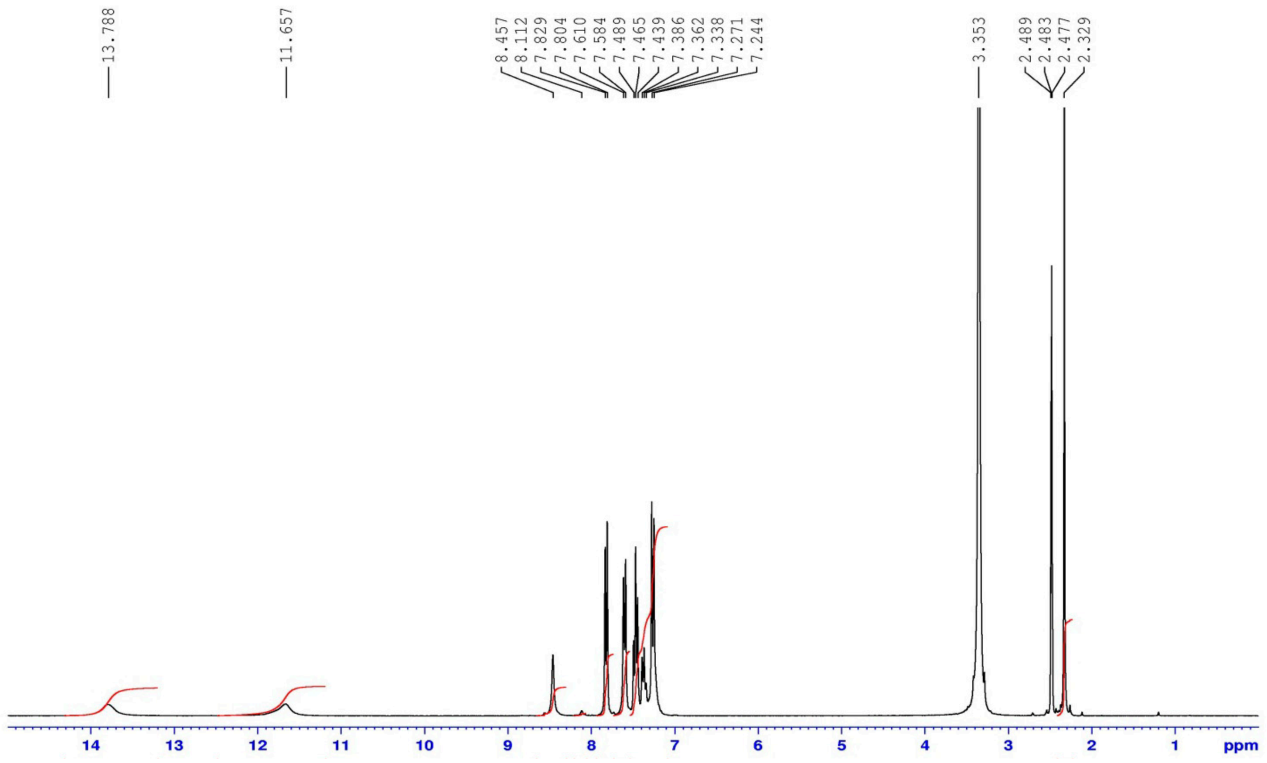

Figure S13. ^{1}H NMR spectrum of **L2**.

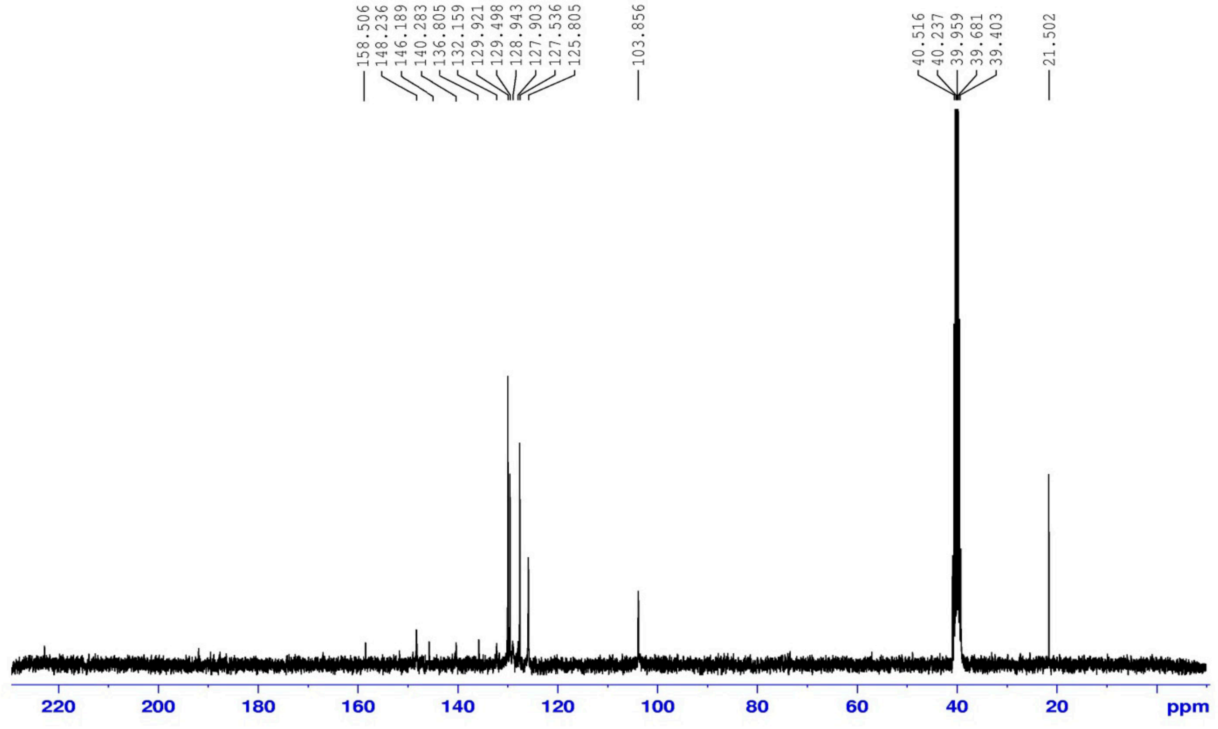

Figure S14. ^{13}C RMN spectrum of **L2**.

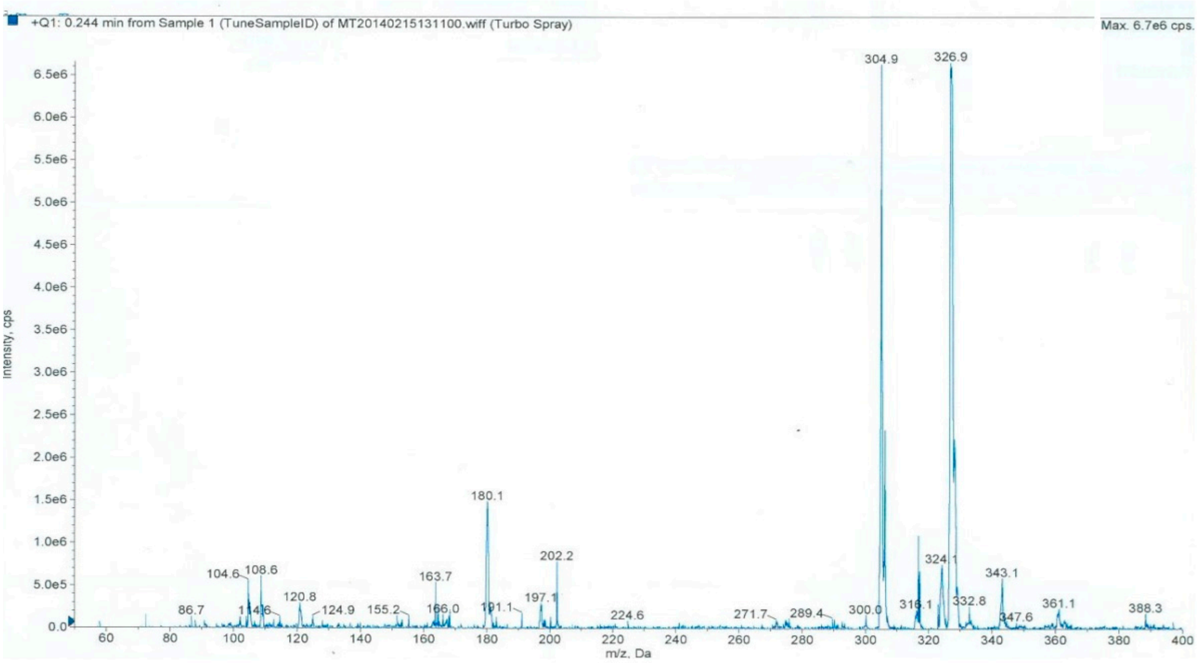

Figure S15. Mass spectrum of **L2**.

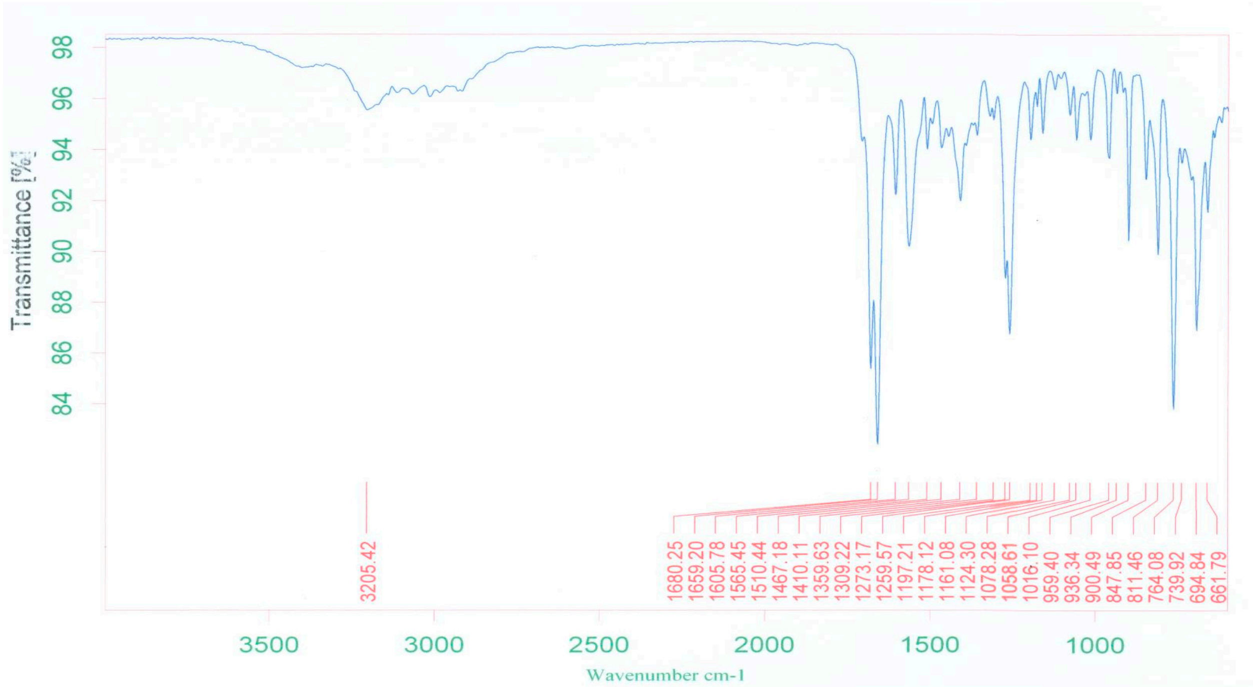

Figure S16. FT-IR spectrum of **L3**.

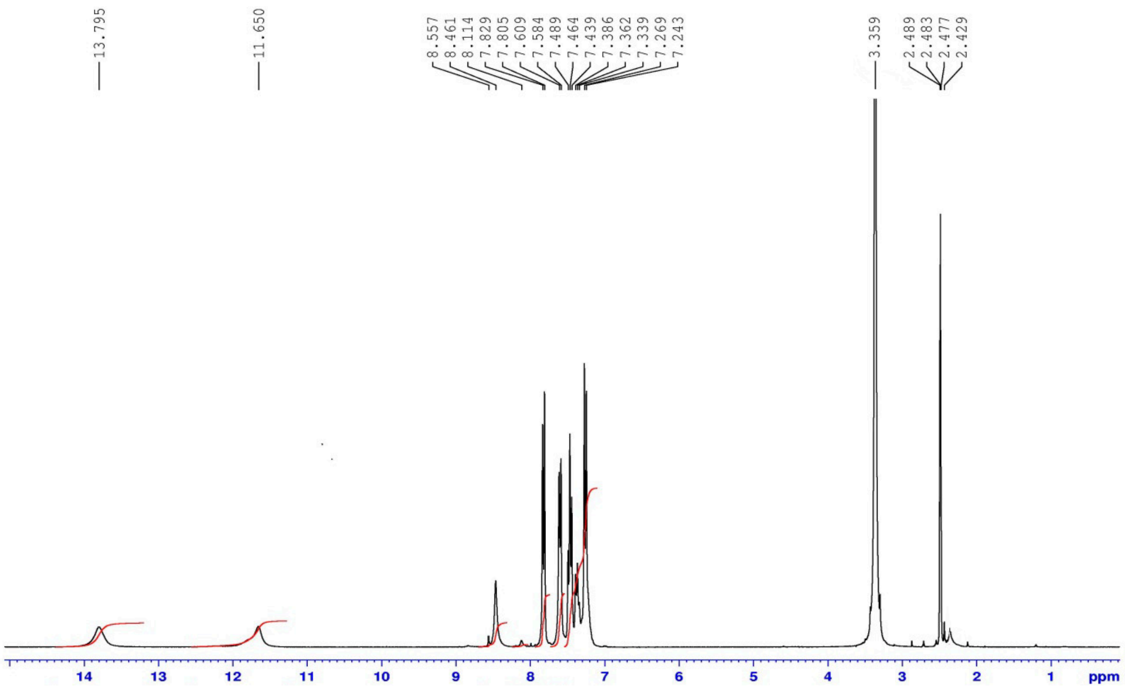

Figure S17. ^{1}H NMR spectrum of **L3**.

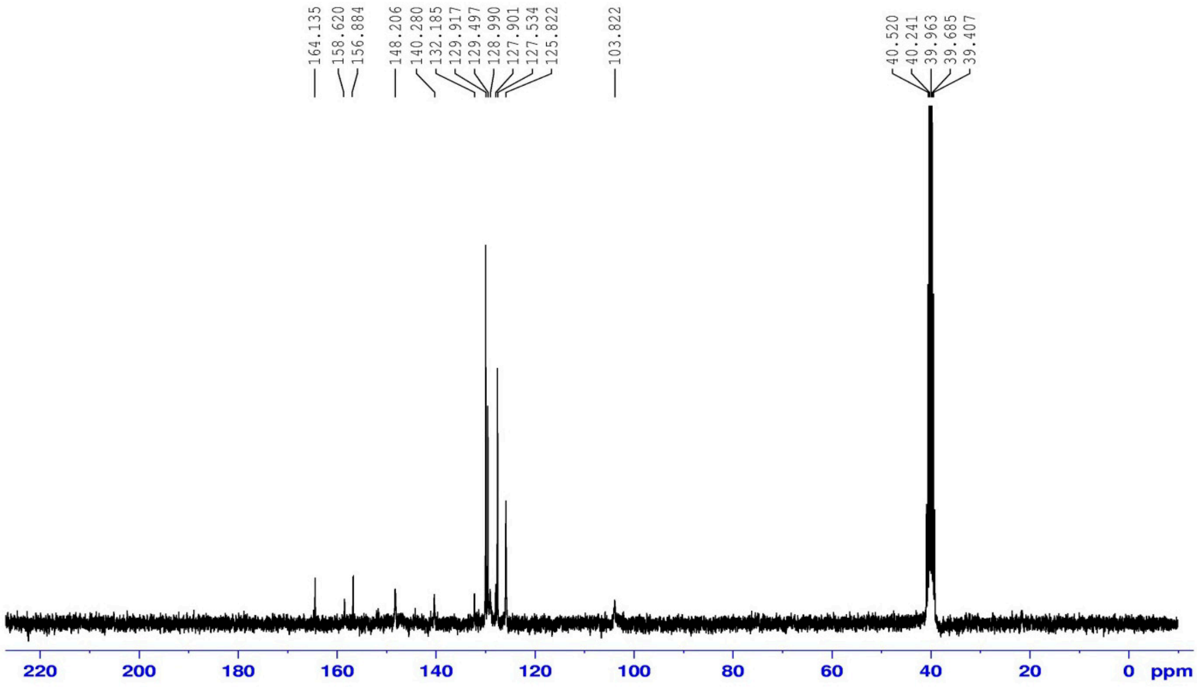

Figure S18. ^{13}C NMR spectrum of **L3**.

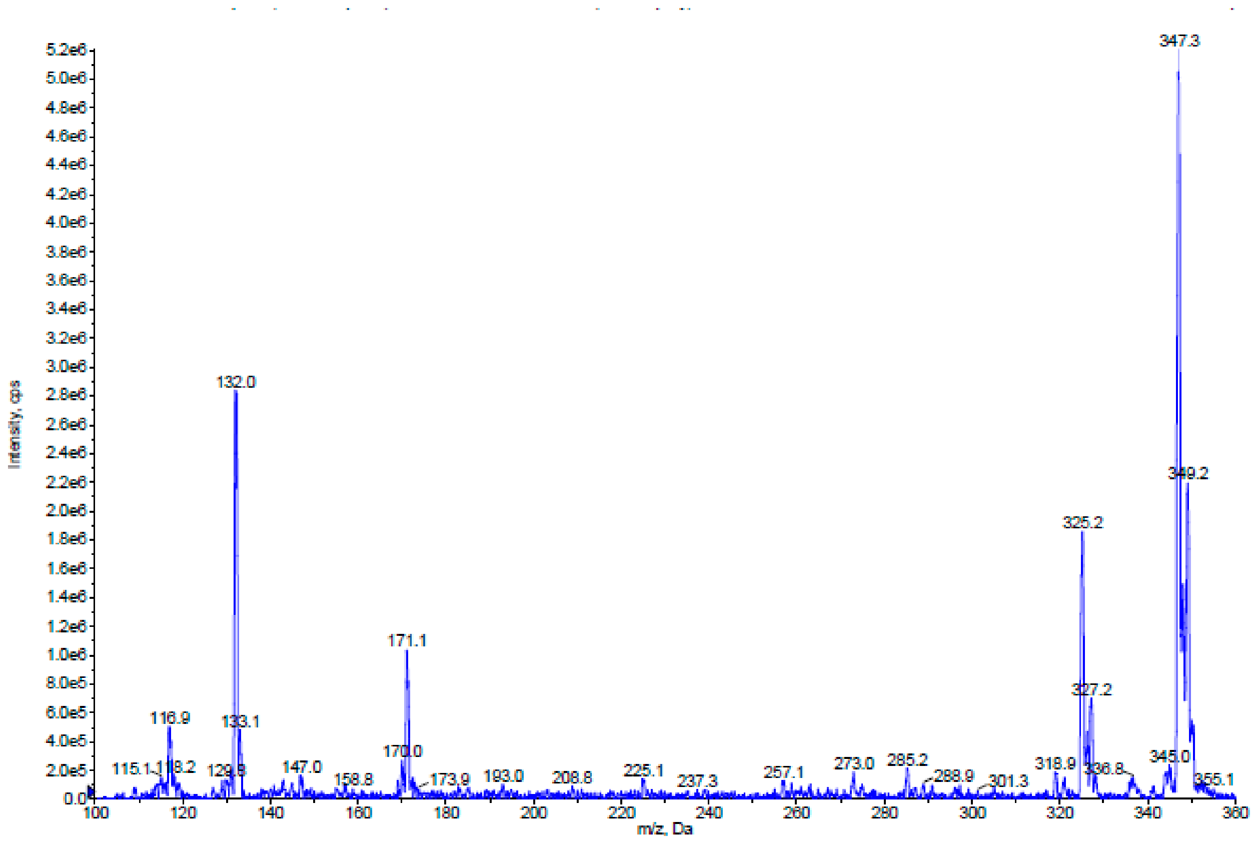

Figure S19. Mass spectrum of **L3**.

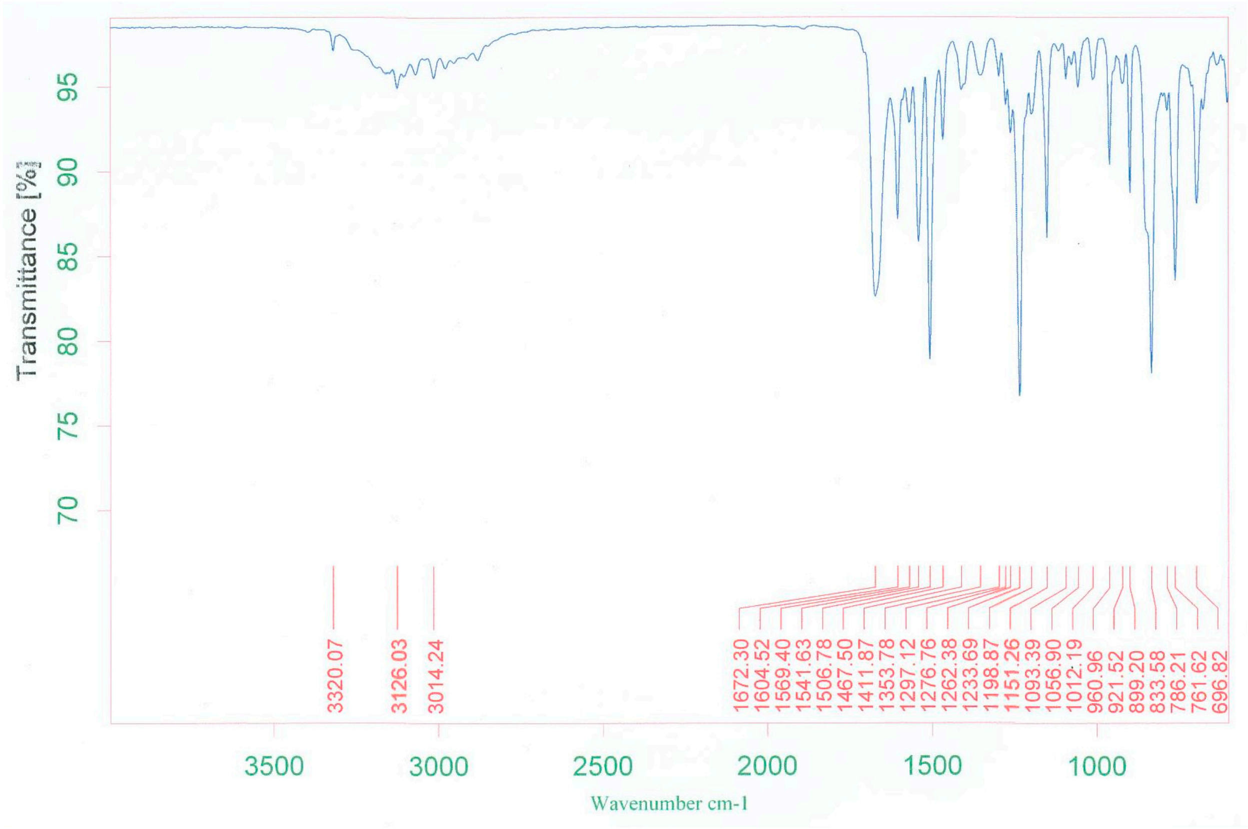

Figure S20. FT-IR spectrum of **L4**.

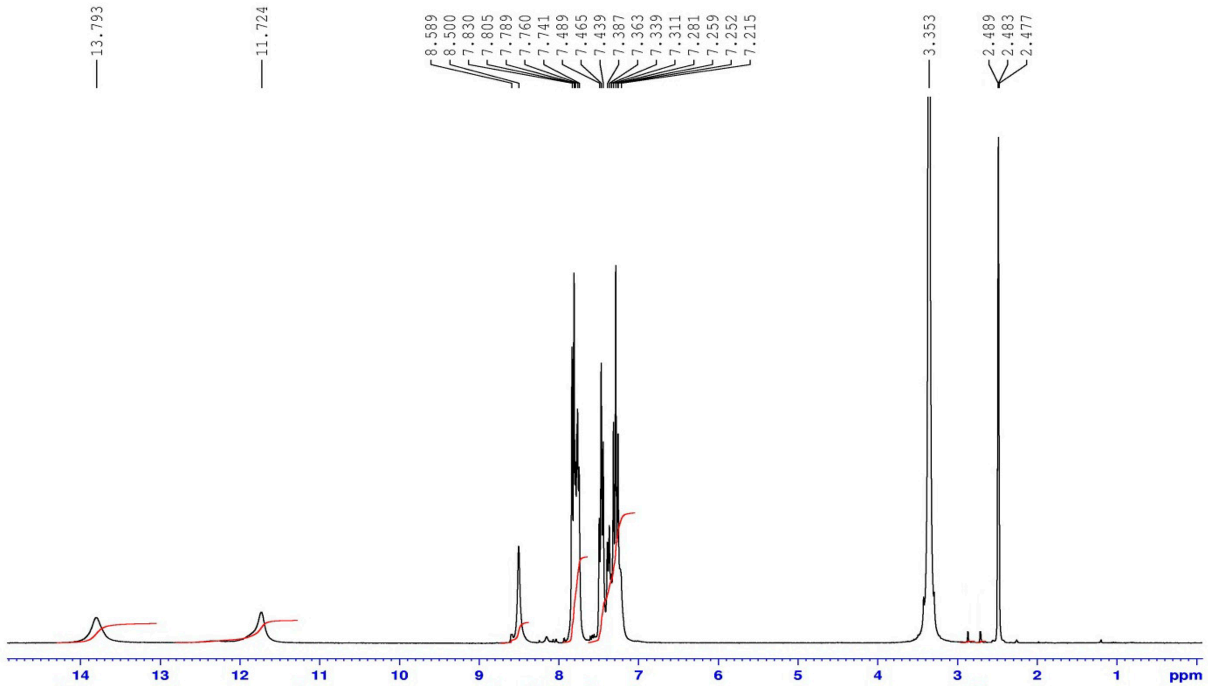

Figure S21. ^{1}H NMR spectrum of **L4**.

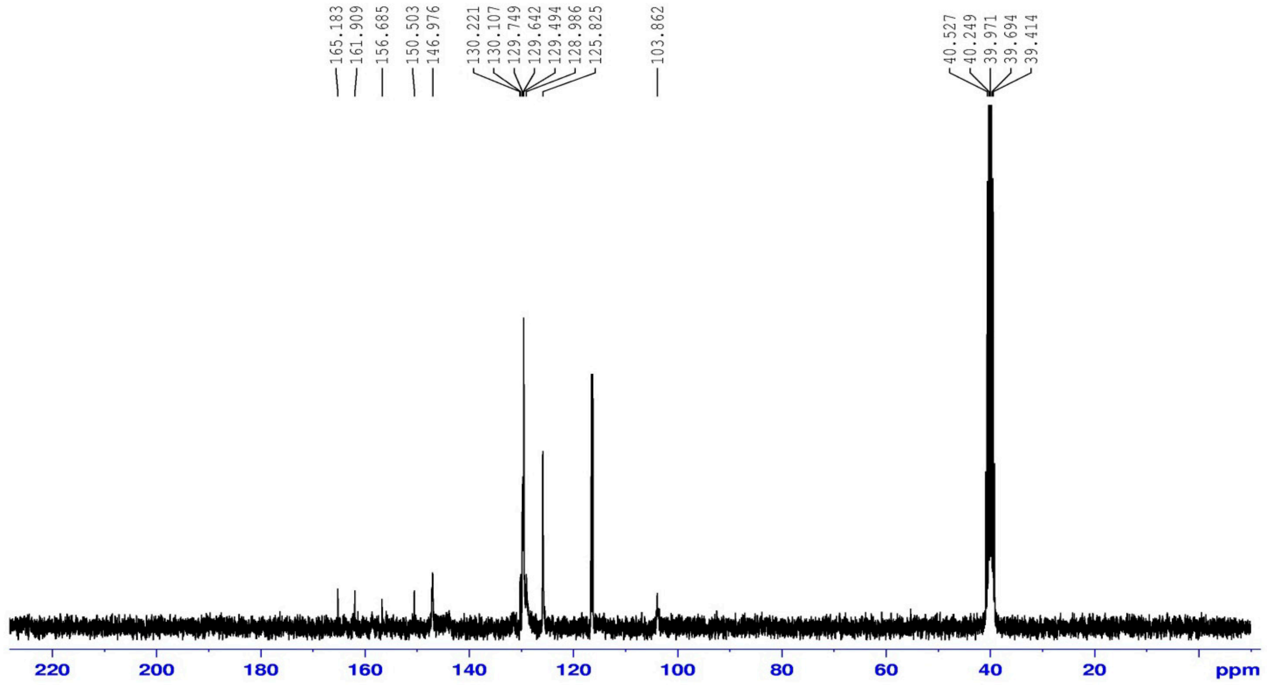

Figure S22. ^{13}C NMR spectrum of **L4**.

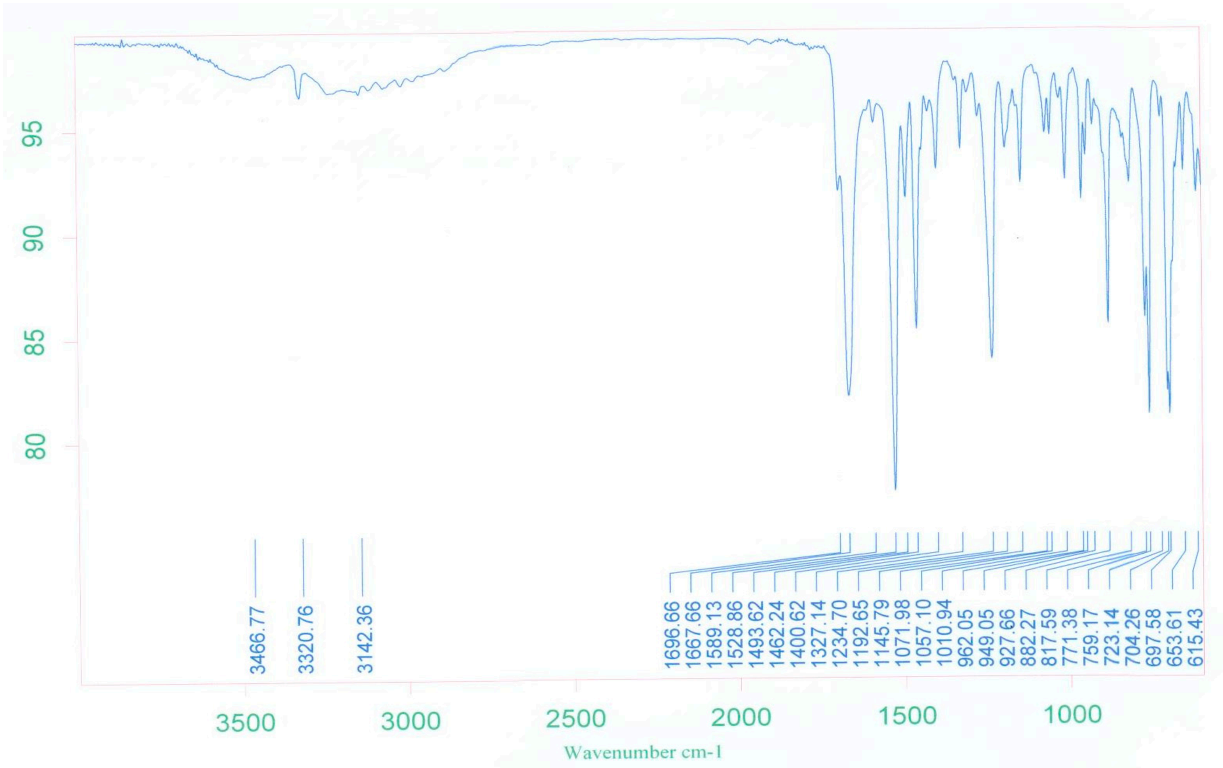

Figure S23. FT-IR spectrum of **L5**.

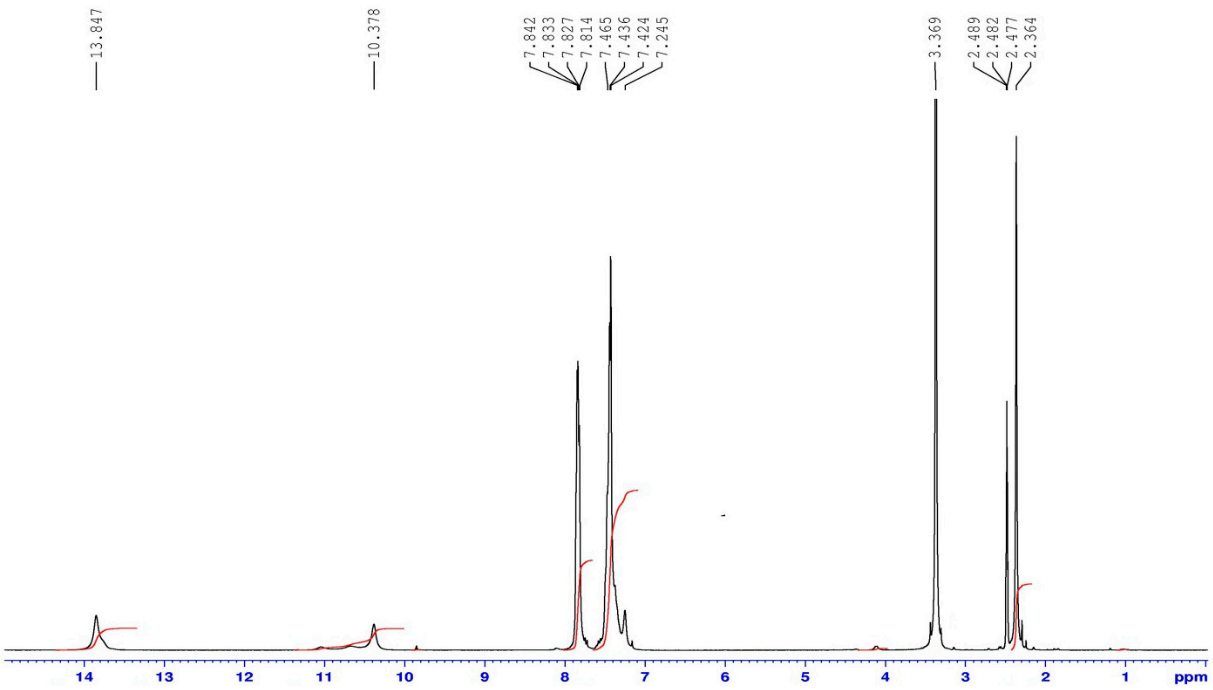

Figure S24. ^{1}H NMR spectrum of **L5**.

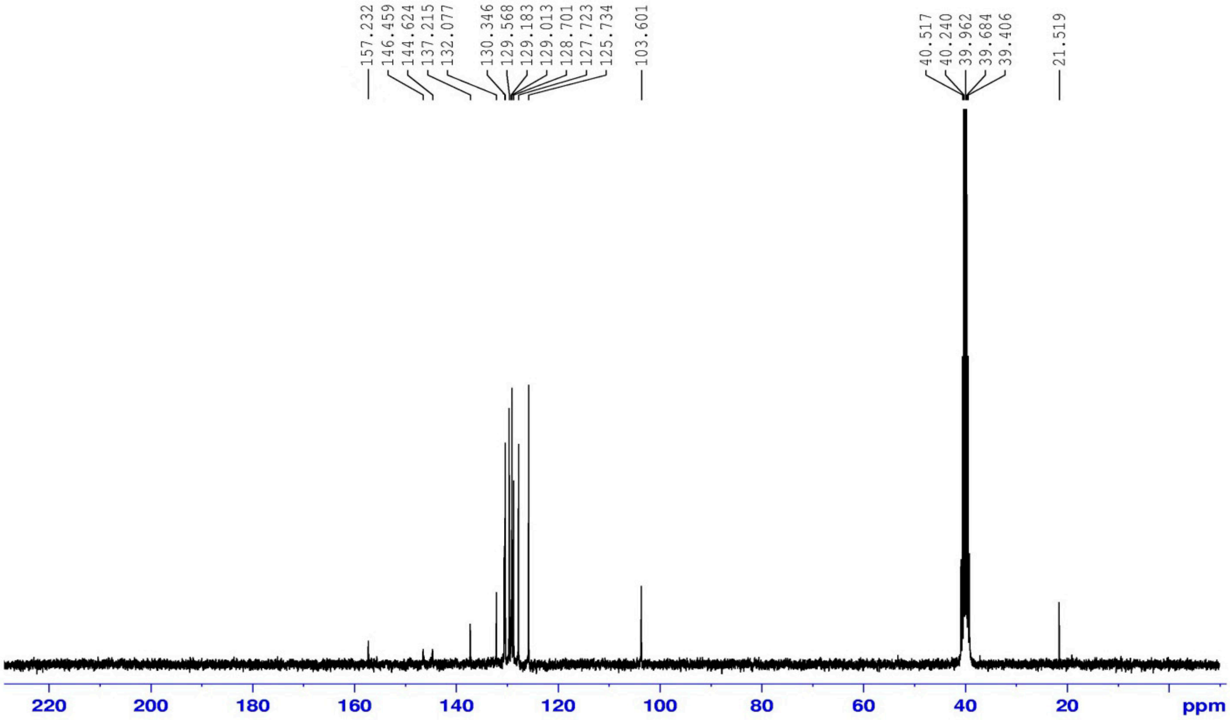

Figure S25. ^{13}C NMR spectrum of **L5**.

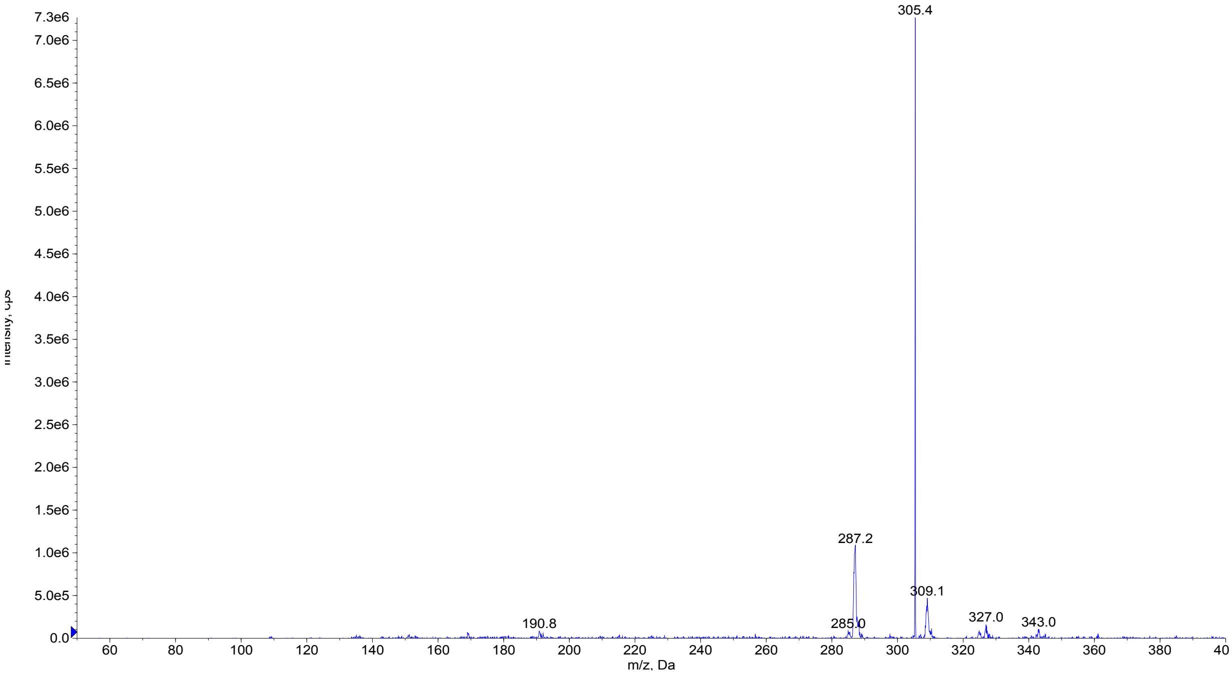

Figure S26. Mass spectrum of **L5**.

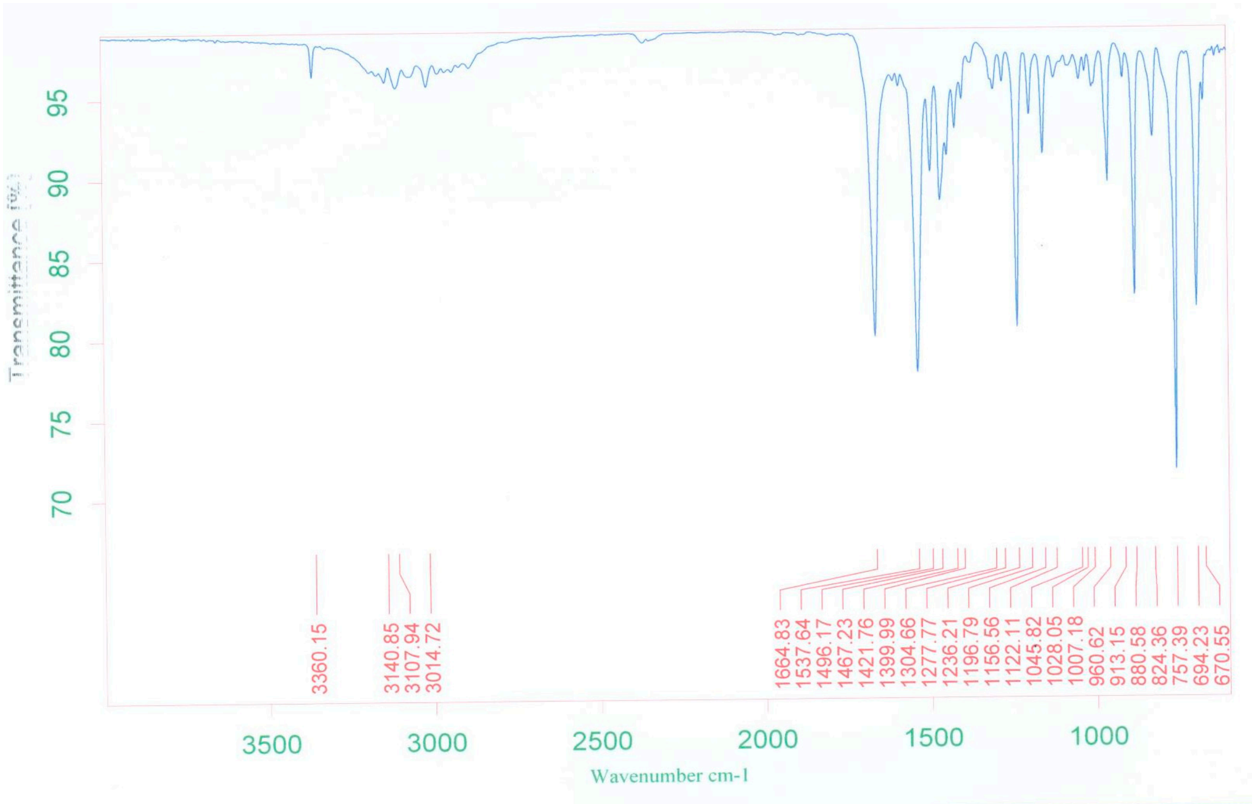

Figure S27. FT-IR spectrum of **L6**.

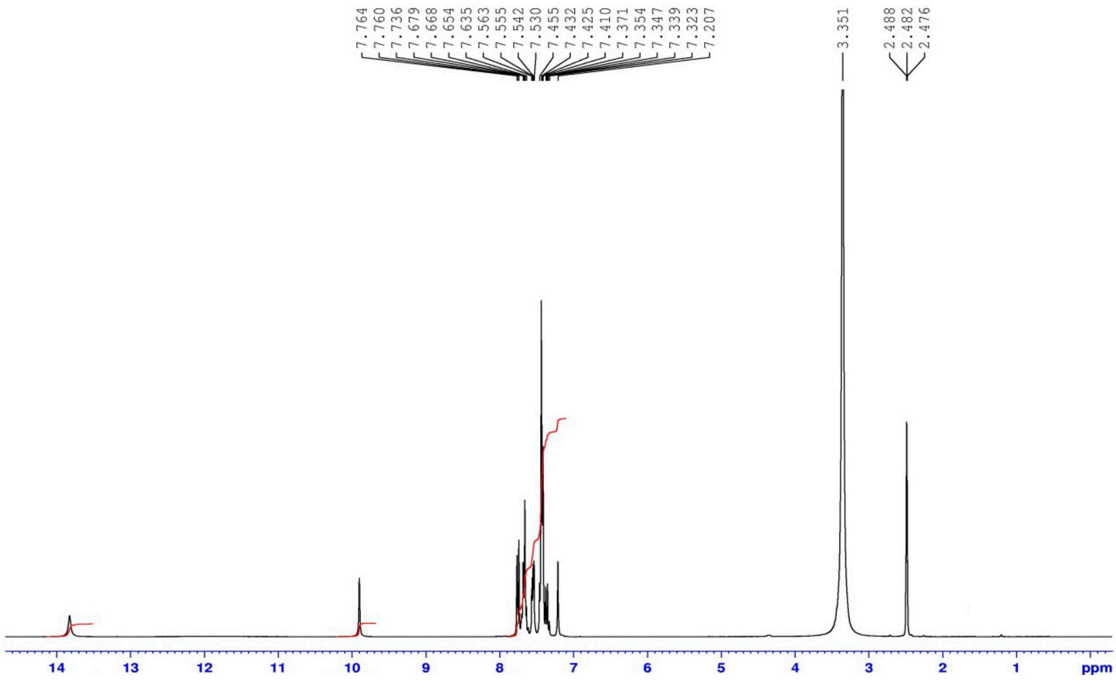

Figure S28. ^{1}H NMR spectrum of **L6**.

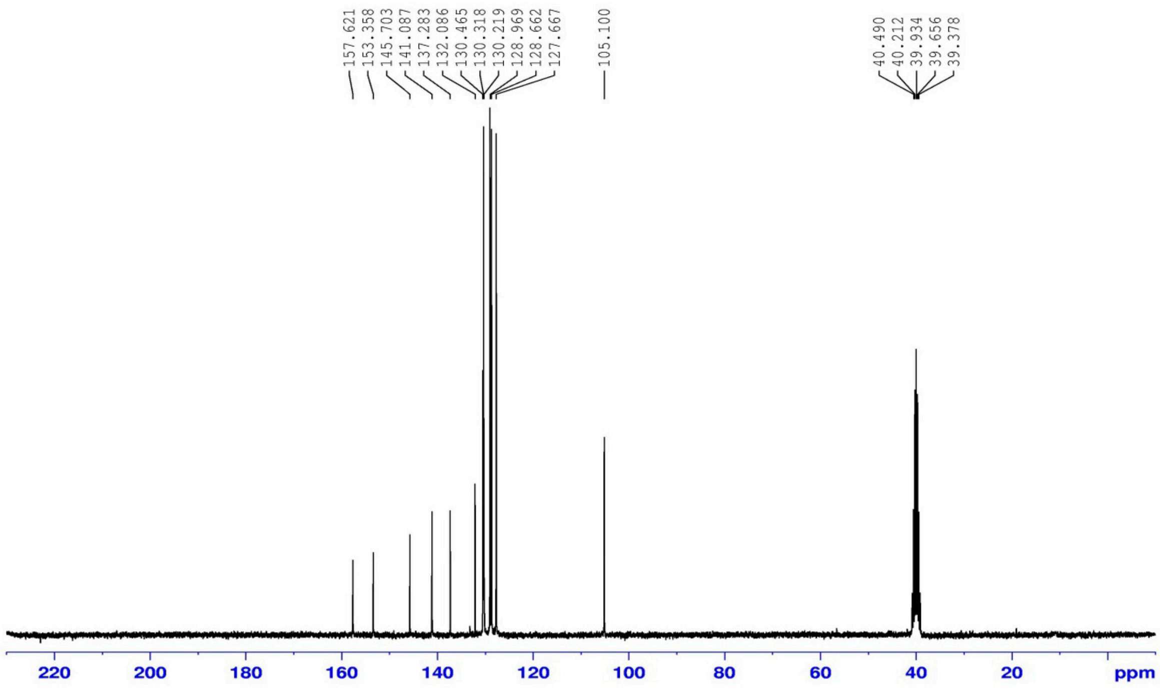

Figure S29. ^{13}C NMR spectrum of **L6**.

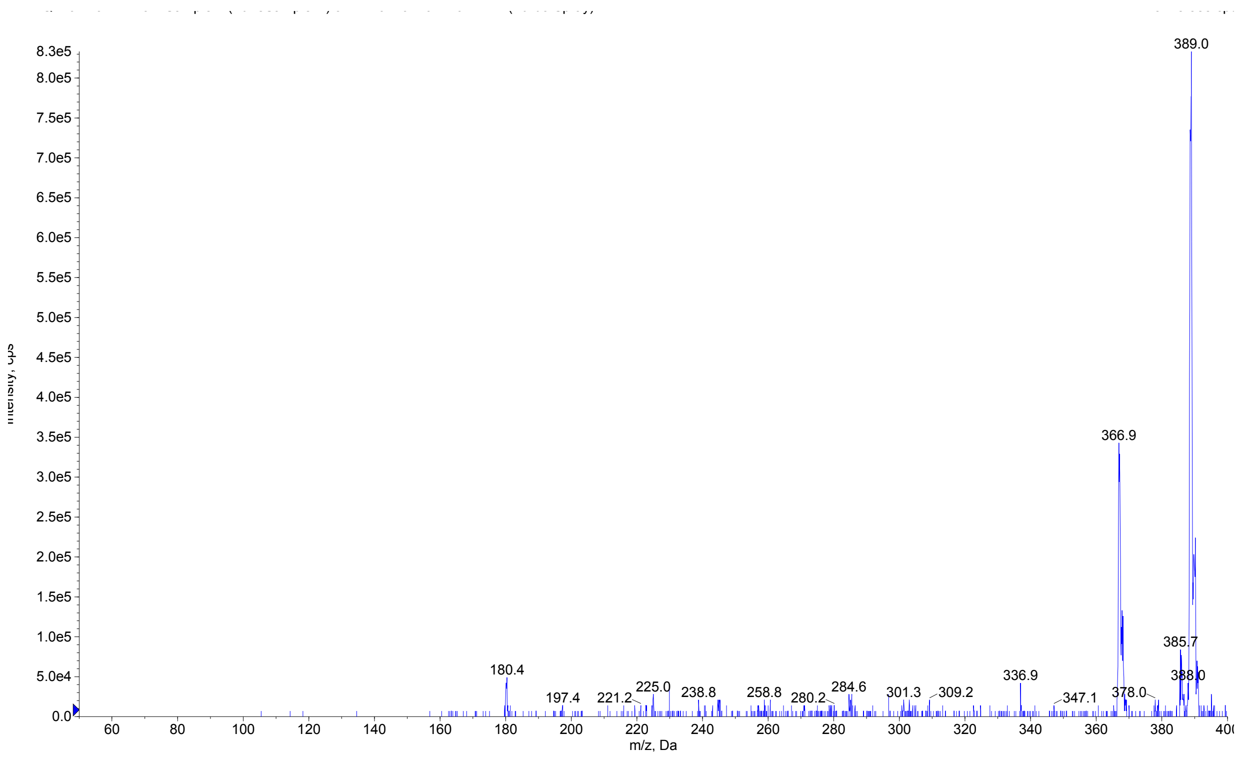

Figure S30. Mass spectrum of **L6**.
